# Environmental impact of diets for dogs and cats

**DOI:** 10.1038/s41598-022-22631-0

**Published:** 2022-11-17

**Authors:** Vivian Pedrinelli, Fabio A. Teixeira, Mariana R. Queiroz, Marcio A. Brunetto

**Affiliations:** School of Veterinary Medicine and Animal Science, University of Sao Paulo, Sao Paulo, Brazil

**Keywords:** Environmental sciences, Environmental impact

## Abstract

Food production is responsible for almost one-quarter of the environmental impact and, therefore, its importance regarding sustainability should not be overlooked. The companion animal population is increasing, and an important part of pet food is composed of ingredients that have a high environmental impact. This study aimed to evaluate the impact of dry, wet, and homemade pet diets on greenhouse gas emission, land use, acidifying emission, eutrophying emissions, freshwater withdrawals, and stress-weighted water use. The wet diets were responsible for the highest impact, and dry diets were the type of diet that least impacted the environment, with a positive correlation between the metabolizable energy provided by animal ingredients and the environmental impact. It is necessary to consider the environmental impact of pet food since it is significant, and the population of pets tends to increase.

## Introduction

Companion animals are considered part of the family, and their population is growing^1^. The three top countries regarding canine population are the U.S. (76.8 mi dogs), Brazil (52.2 mi), and China (27.4 mi), and regarding the feline population the top three countries are the U.S. (58.4 mi), China (53.1 mi), and Brazil (22.1 mi)^2–4^. In Brazil, according to a nationwide census in 2013, the dog population has overcome the number of children^2^. This expansion in the pet population increases the demand for products of this segment, including food^5^. Because pet foods are rich in ingredients of animal origin, and this type of ingredient is known to be responsible for higher gas emissions and land use^6,7^, it is important to consider their impact on the environment.

A meta-analysis on the impact of food, which included 38,700 farms and 119 countries, observed that food production is responsible for 26% of total anthropogenic greenhouse gas emission (GHG)^7^. According to the authors, animal production, including fish, is responsible for 31% of GHG, and crops are responsible for 27%. The land use corresponds to 24% of emissions, of which 16% are related to animal production and 8% to crops. The Food and Agriculture Organization (FAO) estimates that 50% of the habitable land and 70% of freshwater withdrawals are used for agriculture^8^.

GHGs are gas substances that constitute the atmosphere and can be natural or anthropogenic, which absorb radiation emitted by the terrestrial surface. They prevent the loss of heat to space, keeping the terrestrial surface potentially warmer and, therefore, can cause alterations to the atmosphere balance. Some of the GHGs are carbon dioxide (CO_2_), methane (CH_4_), nitrous oxide (N_2_O), ozone (O_3_), and water vapor. The emission of carbon dioxide equivalents (CO_2_eq) represents the mass of CO_2_ that causes the same radiative forcing of a determined GHG mass over the same period and is a measure that comprehends all the GHGs^9^. For most of the foods, the highest percentage of GHG emission results from the change in the soil, which is caused by deforestation and carbon composition of the soil, along with fertilizers. Together, they can represent about 80% of the CO_2_eq of foods^7,9,10^.

Land use is another tool to estimate environmental impact. It is an important parameter to indicate if a region can support the production of food. For example, livestock accounts globally for 77% of farming land and produces only 37% of total protein^7^. Other indicators of environmental impact are acidifying emissions (as sulfur dioxide equivalent emission), eutrophying emissions (as phosphate equivalent emissions), freshwater withdrawals, and stress-weighted water use^7^.

Little is known regarding the impact of feeding canine and feline populations. A study^11^ observed that the ecological footprint (or pawprint) of the Chinese population of dogs and cats is equivalent to 70 to 245 million Chinese citizens, depending on the size of the animal and diet consumed. Another study conducted in Japan^12^ observed that the ecological pawprint of a dog can be similar to that of one Japanese citizen. In the U.S., a study^13^ observed that the canine population was responsible for between 25 and 30% of the animal production impact regarding land use, water, and fossil fuels. A recent study^14^ estimated the global pawprint of pet food based on dry diets from the U.S. and observed that pet food could be responsible for up to 2.9% of CO_2_eq emission and up to 1.2% of agricultural land use. All of these studies, however, used different methods to evaluate the diet composition, considering either hypothetical diets or only dry diets.

Therefore, the aim of this study was to evaluate the environmental impact of different types of diets for dogs and cats in Brazil, since it is among the top countries regarding canine and feline population and is representative in a global environmental impact scenario.

## Results

### Profile of diets

A total of 938 diets, 618 for dogs (316 commercial dry, 81 commercial wet, 139 commercial homemade, and 82 homemade from websites) and 320 for cats (180 commercial dry, 104 commercial wet, 11 commercial homemade, and 26 homemade from websites) were included in the present study (see the supplementary materials). A total of 212 ingredients were found at diet label or websites, of which 46.2% of animal sources (n = 98/212) and 53.8% of vegetable sources (n = 114/212). Ingredients of commercial wet and dry diets were 49.5% of animal sources (n = 47/95 ingredients listed) and 50.5% of vegetable sources (n = 48/95), whereas the ingredients of homemade diets (commercial and website) were 45.3% of animal sources (n = 68/150 ingredients listed) and 54.7% of vegetable sources (n = 82/150). The five most common ingredients in commercial dry and wet diets were poultry by-product meal (used in 488 diets), poultry fat (in 478 diets), whole cornmeal (in 355 diets), broken rice (in 342 diets), and beet pulp (in 316 diets). The five most common ingredients in commercial or internet homemade diets were cooked carrot (in 134 diets), cooked squash (in 79 diets), cooked sweet potato (in 77 diets), cooked zucchini (in 74 diets), and cooked chayote (in 73 diets) (Table S3).

### Macronutrient profile

Dry diets for both dogs and cats presented the highest metabolizable energy (kcal/g) (p < 0.001) and nitrogen-free extract (NFE) content (g/1000 kcal) (p < 0.001). As for protein content (g/1000 kcal), wet diets for dogs presented the highest amounts, followed by homemade diets (p < 0.001), and wet and website homemade diets for cats had the highest amounts (p < 0.001). Regarding fat content (g/1000 kcal), wet diets for dogs contained the highest amounts (p < 0.001), whereas for cats the wet diets were higher in fat than dry diets (p < 0.001).

The profile for metabolizable energy, crude protein, crude fat, and nitrogen-free extract of each category of diet for dogs can be observed in Fig. S1, and for cats in Fig. S2.

### Nutrient and energy sources

**Figure 1:**
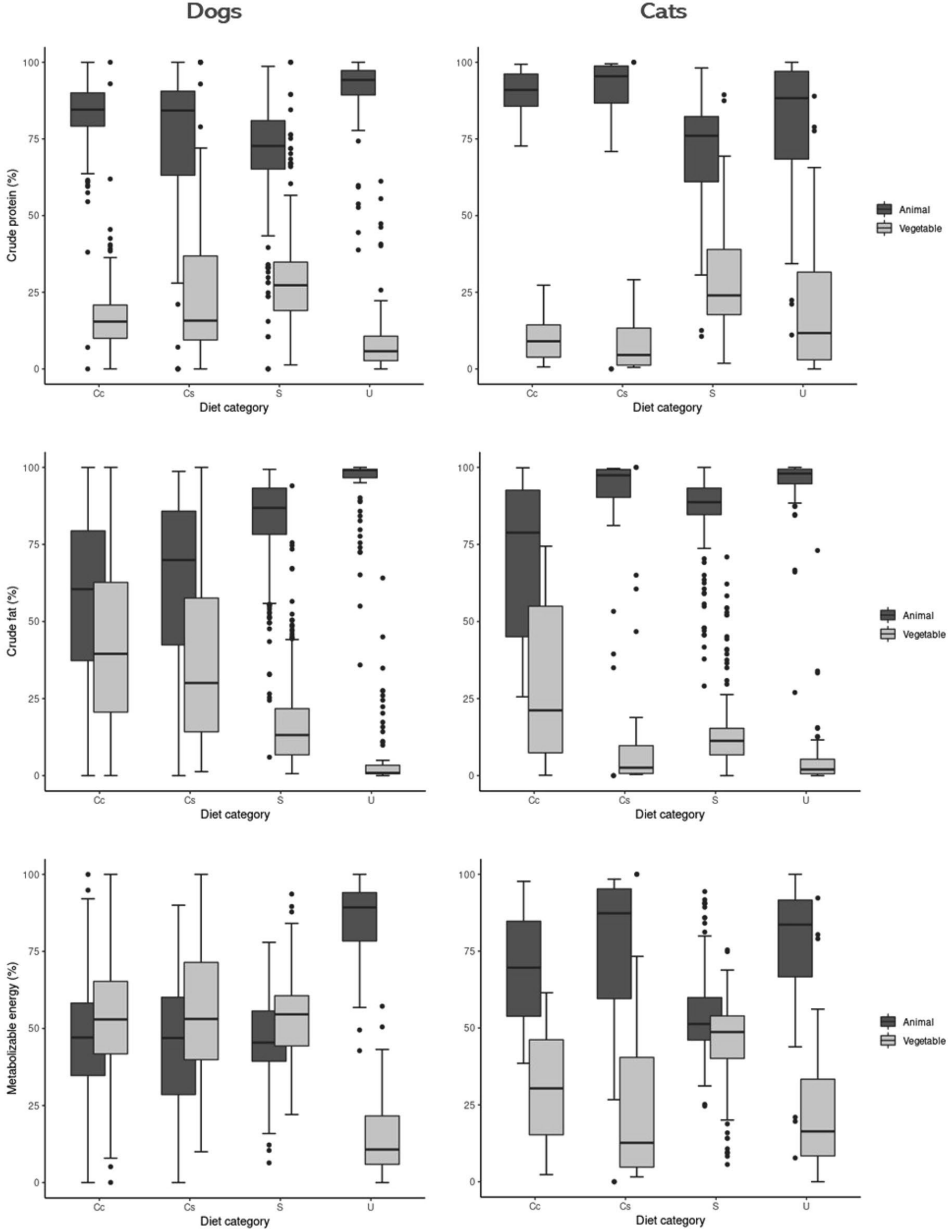
Boxplots of the distribution of percentages of crude protein, crude fat, and amount of metabolizable energy provided by either animal or vegetable origin for each type of diet. Diet category: *Cc* homemade diets, *Cs* website homemade diets, *S* dry diets, and *U* wet diets. Plots of the same variable that have different letters differed (p < 0.05) according to the multiple comparison test between groups.

The protein, fat, and metabolizable energy sources, whether of animal or vegetable origin, were evaluated and the results are presented in Tables S1 and S2 and Fig. 1. The median percentages of protein and fat from animal origin were significantly higher for all of the types of diets, for both dogs (p < 0.001) and cats (p ≤ 0.03), and the metabolizable energy provided by animal ingredients was higher for all diet types for cats (p < 0.001). The metabolizable energy provided by animal sources for dogs was only higher for dry (p < 0.001) and wet diets (p < 0.001), with no difference in commercial (p = 0.1) or website (p = 0.09) homemade recipes.

### Environmental impact estimate

**Figure 2:**
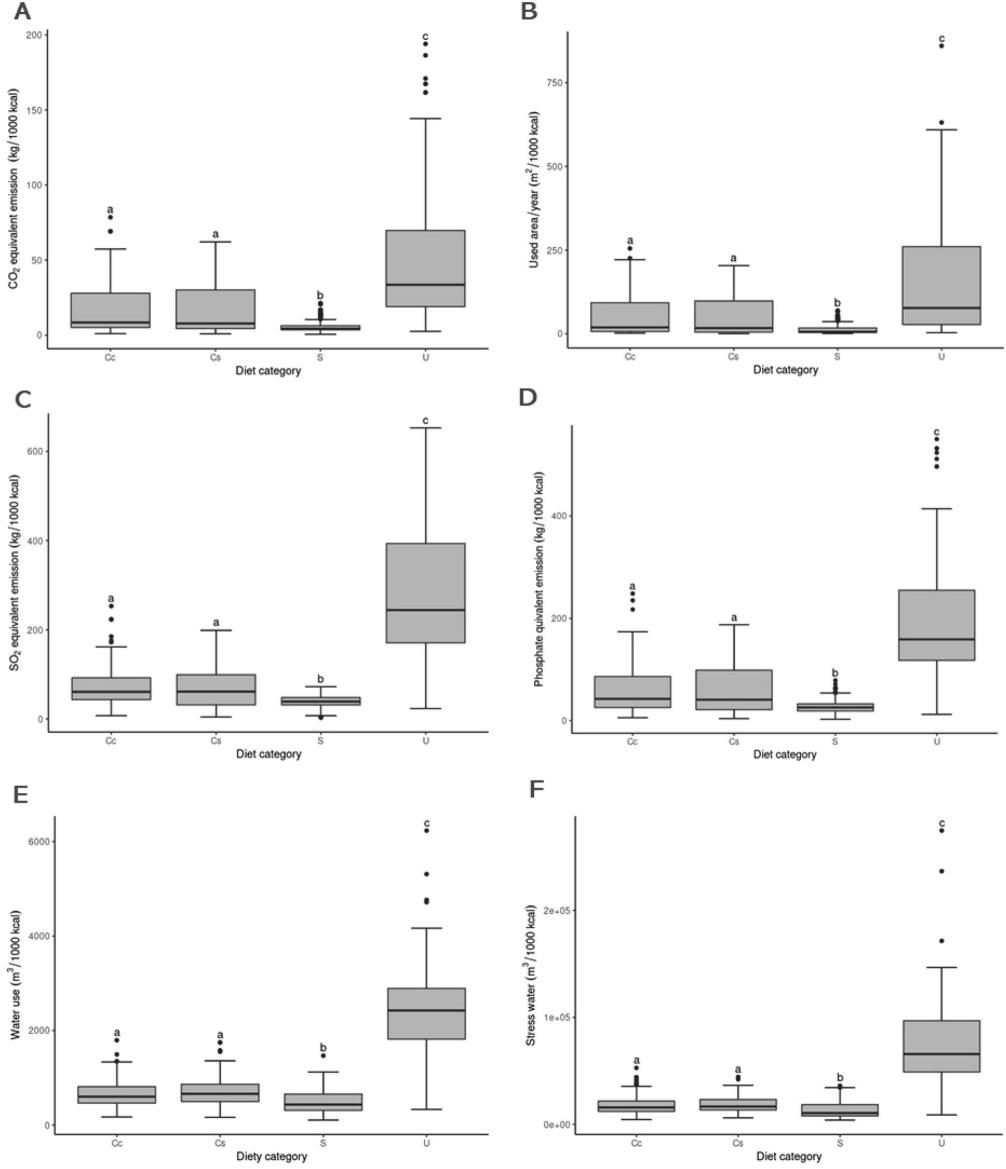
Boxplots of the estimated environmental impact per 1000 kcal of diets for dogs according to the type of diet for the variables carbon dioxide equivalent emission, land use, acidifying emission, eutrophying emission, freshwater withdrawal, and stress-weighted water use. Plots of the same variable that have different letters differed (p < 0.05) according to the multiple comparison test between groups. Diet category: *Cc* homemade diets, *Cs* website homemade diets, *S* dry diets, and *U* wet diets. Plots of the same variable that have different letters differed (p < 0.05) according to the multiple comparison test between groups.

**Figure 3:**
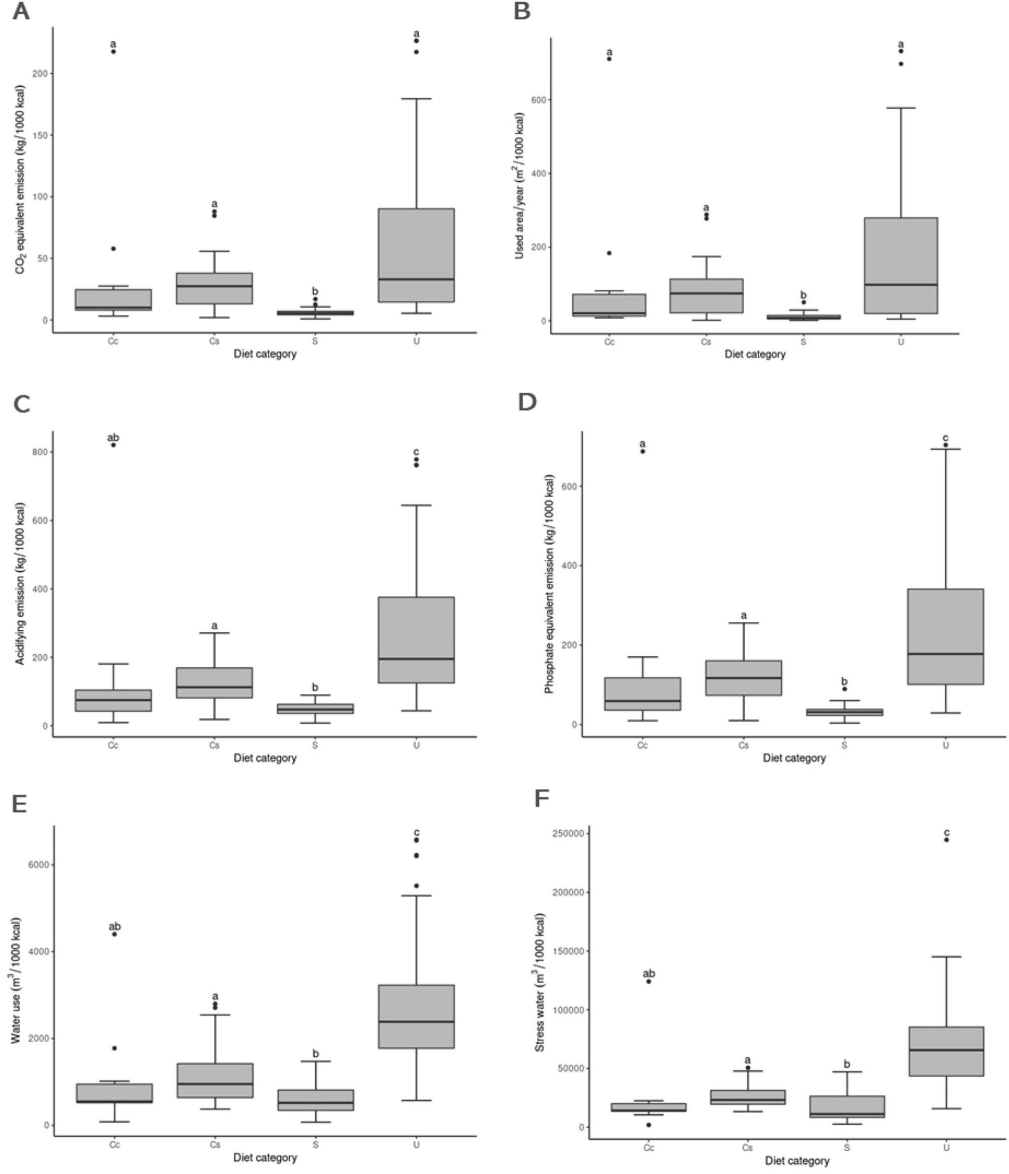
Boxplots of the estimated environmental impact per 1000 kcal of diets for dogs according to the type of diet for the variables carbon dioxide equivalent emission, land use, acidifying emission, eutrophying emission, freshwater withdrawal, and stress-weighted water use. Plots of the same variable that have different letters differed (p < 0.05) according to the multiple comparison test between groups. Diet category: *Cc* homemade diets, *Cs* website homemade diets, *S* dry diets, and *U* wet diets. Plots of the same variable that have different letters differed (p < 0.05) according to the multiple comparison test between groups.

For all of the variables of environmental impact evaluated, wet diets represented a significantly greater impact on the environment, for both dogs (Fig. 2) and cats (Fig. 3). In most cases, dry diets were responsible for less environmental impact than the other types of diets. Regarding homemade diets, the environmental impact was intermediary between wet and dry diets, except for acidifying emissions, freshwater withdrawals, and stress-weighted water use in diets for cats, in which they were similar between dry diets and commercial homemade diets.

**Figure 4:**
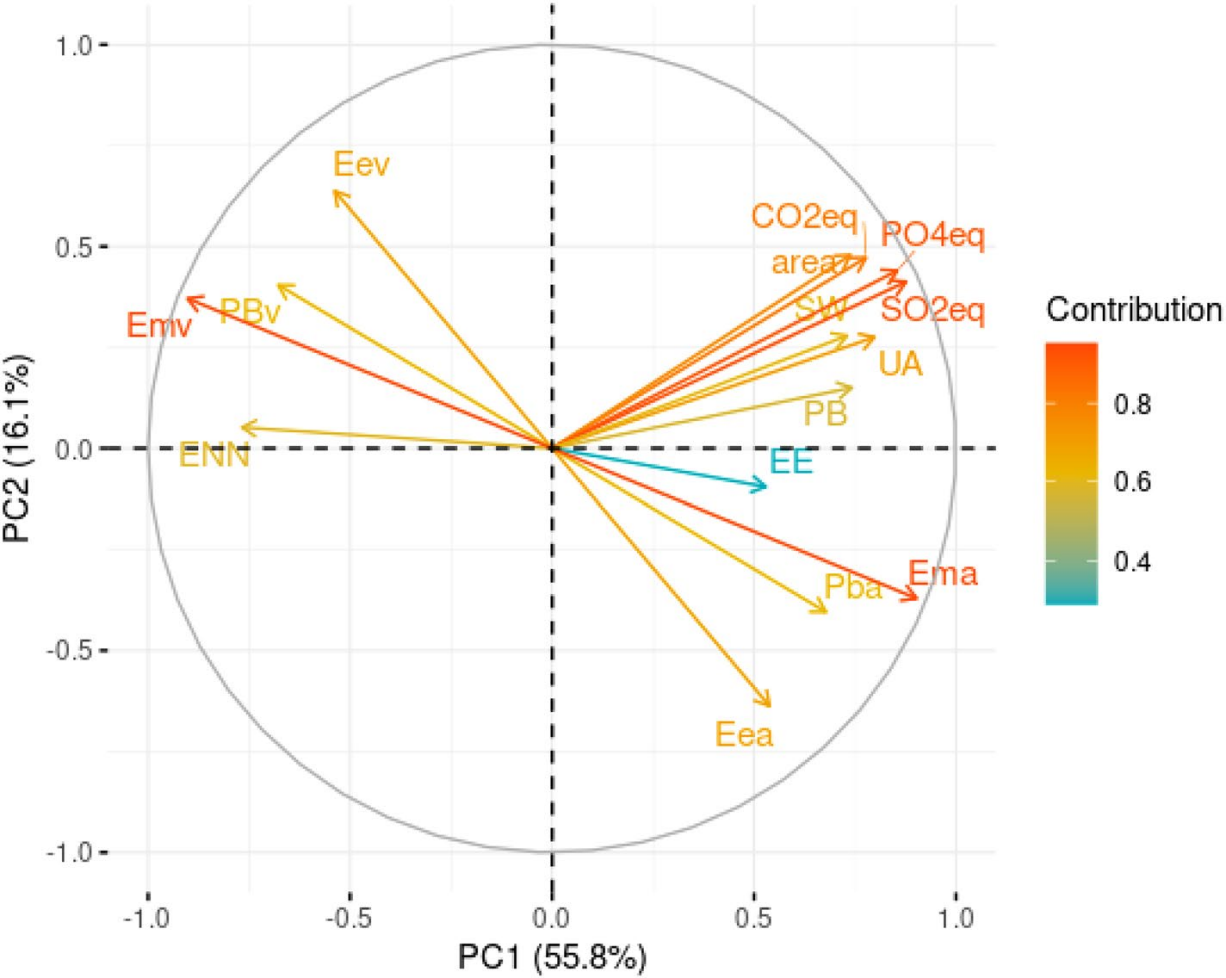
Principal component analysis (PCA) of first principal component (PC1) versus second principal component (PC2) of diets for dogs. *ENN*  nitrogen-free extract, *PBv* protein from vegetable sources, *Emv* metabolizable energy from vegetable sources, *Eev* fat from vegetable sources, *CO2eq* carbon dioxide equivalent, *area* land use, *PO4eq *phosphate equivalent, *SW* stress-weighted water usage, *SO2eq* sulphur oxide equivalent, *UA* freshwater withdrawals, *PB* protein content, *EE* fat content, *Ema* metabolizable energy from animal sources, *Pba* protein from animal sources, *Eea* fat from animal sources.

To summarize the data set information and better explore the contributions of different diet variables evaluated on the environmental impact parameters studied. Figure 4 shows the results of the principal component analysis (PCA) of diets for dogs, considering the first (PC1) and second (PC2) principal components, responsible for 71.9% of data variance for dogs (Fig. S3). For variables regarding diets for dogs, the metabolizable energy was one of the characteristics that were most responsible for the horizontal dispersion of the data, followed by sulfur oxide equivalent (SO_2_eq) and phosphate equivalent (PO_4_^3−^eq). According to the results of the PCA, the higher the metabolizable energy of animal origin, the higher the environmental impact measured with PO_4_^3-^eq and SO_2_eq. The variables that influenced the vertical dispersion the most were the fat from both animal and vegetable origin, followed by land use and CO_2_eq, which have the same direction as the vegetable fat, which suggests that the higher the fat from vegetable origin, the higher the impact measured with CO_2_eq and land use, and an inverse correlation with fat from animal origin. From the PC1, it can also be observed that metabolizable energy, fat, and protein from vegetable origin, as well as NFE, had an inverse relationship with the variables of environmental impact.

**Figure 5:**
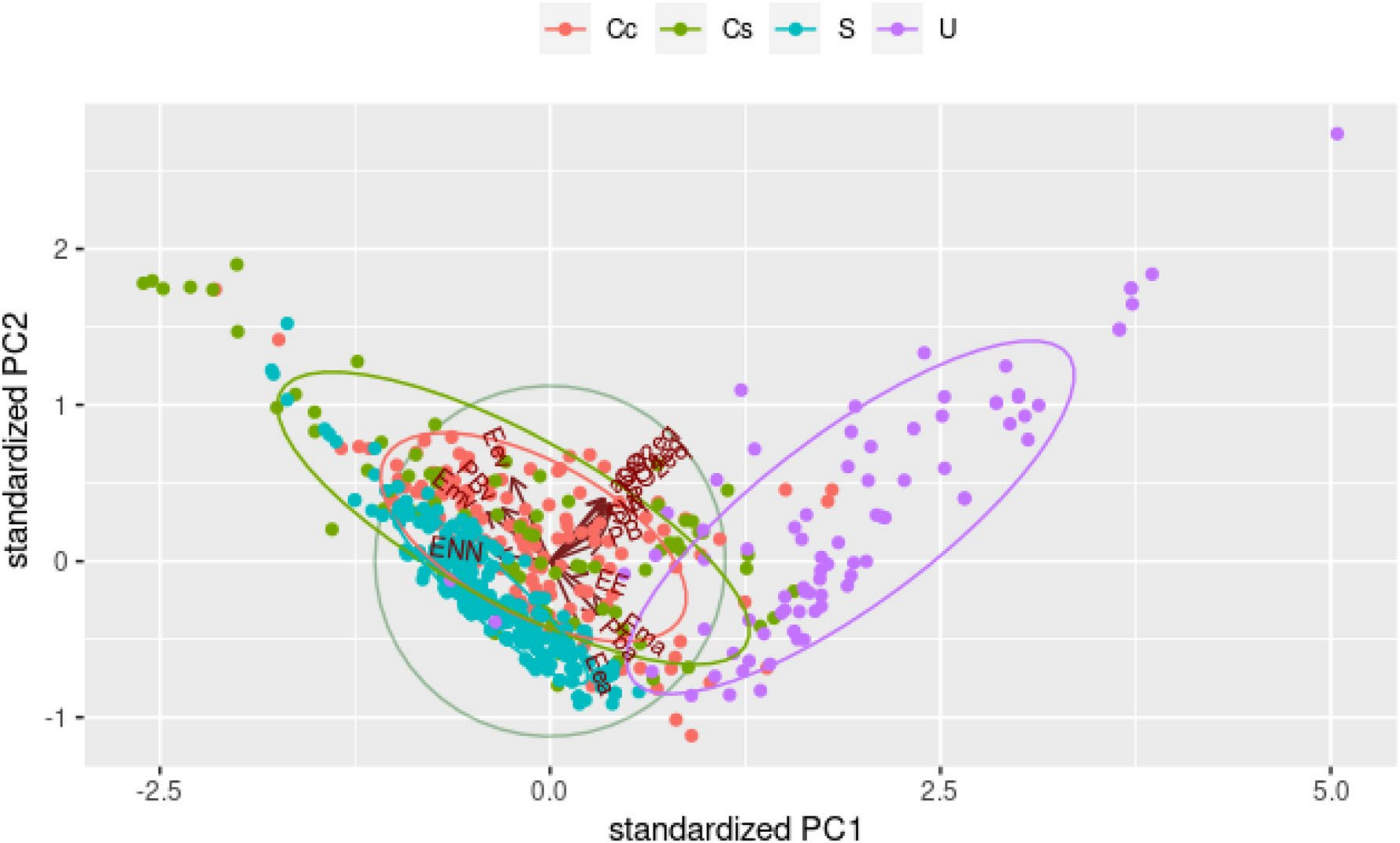
Biplot of standardized first (PC1) and second principal components (PC2) with observations of diets for dogs. *Cc *homemade diets, *Cs* website homemade diets, *S* dry diets, *U* wet diets, *ENN* nitrogen-free extract, *PBv* protein from vegetable sources, *Emv* metabolizable energy from vegetable sources, *Eev* fat from vegetable sources, *CO2eq* carbon dioxide equivalent, *area* land use, *PO4eq* phosphate equivalent, *SW* stress-weighted water usage, *SO2eq* sulphur oxide equivalent, *UA* freshwater withdrawals, *PB* protein content, *EE* fat content, *Ema* metabolizable energy from animal sources, *Pba* protein from animal sources, *Eea* fat from animal sources.

Wet diets were differentiated from the other categories of diets and correlated to an increased environmental impact (Fig. 5).

**Figure 6:**
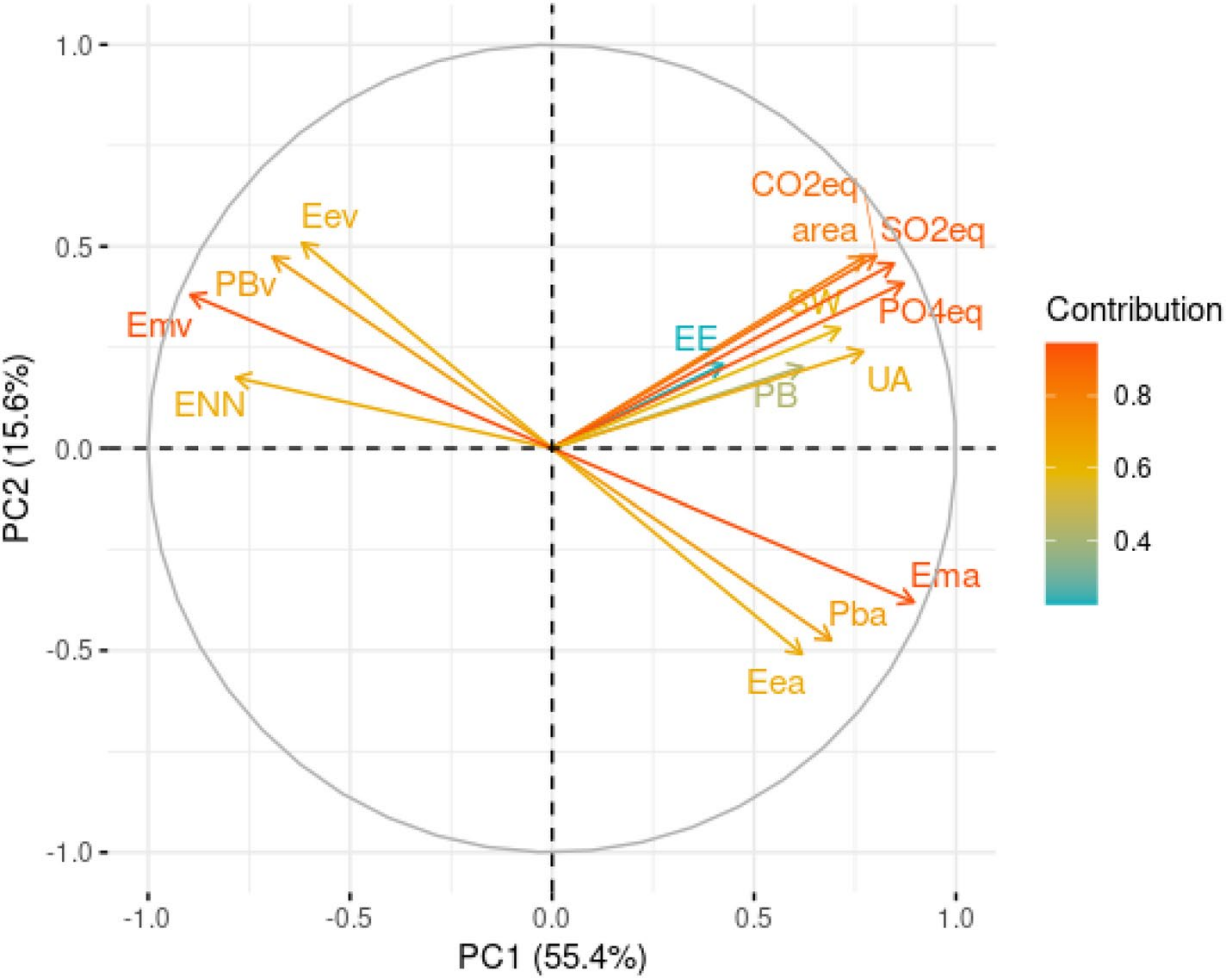
Principal component analysis (PCA) of first principal component (PC1) versus second principal component (PC2) of diets for cats. *ENN* nitrogen-free extract, *PBv* protein from vegetable origin, *Emv *metabolizable energy from vegetable origin, *Eev* fat from vegetable origin, *CO2eq* carbon dioxide equivalent, *area* land use, *PO4eq *phosphate equivalent, *SW* stress-weighted water usage, *SO2eq* sulphur oxide equivalent, *UA* freshwater withdrawals, *PB* protein content, *EE* fat content, *Ema *metabolizable energy from animal sources, *Pba* protein from animal sources, *Eea* fat from animal sources.

Figure 6 shows the results of the PCA of diets for cats, considering PC1 and PC2, responsible for 71.0% of data variance for cats (Fig. S4). The results are very similar to those of diets for dogs, with a correlation between the metabolizable energy of animal origin and the environmental impact measured with PO_4_^3-^eq and SO_2_eq. Regarding the PC1, as it was observed in dogs, the metabolizable energy, fat, and protein from vegetable sources, as well as NFE, presented an inverse relationship with the variables of environmental impact.

**Figure 7:**
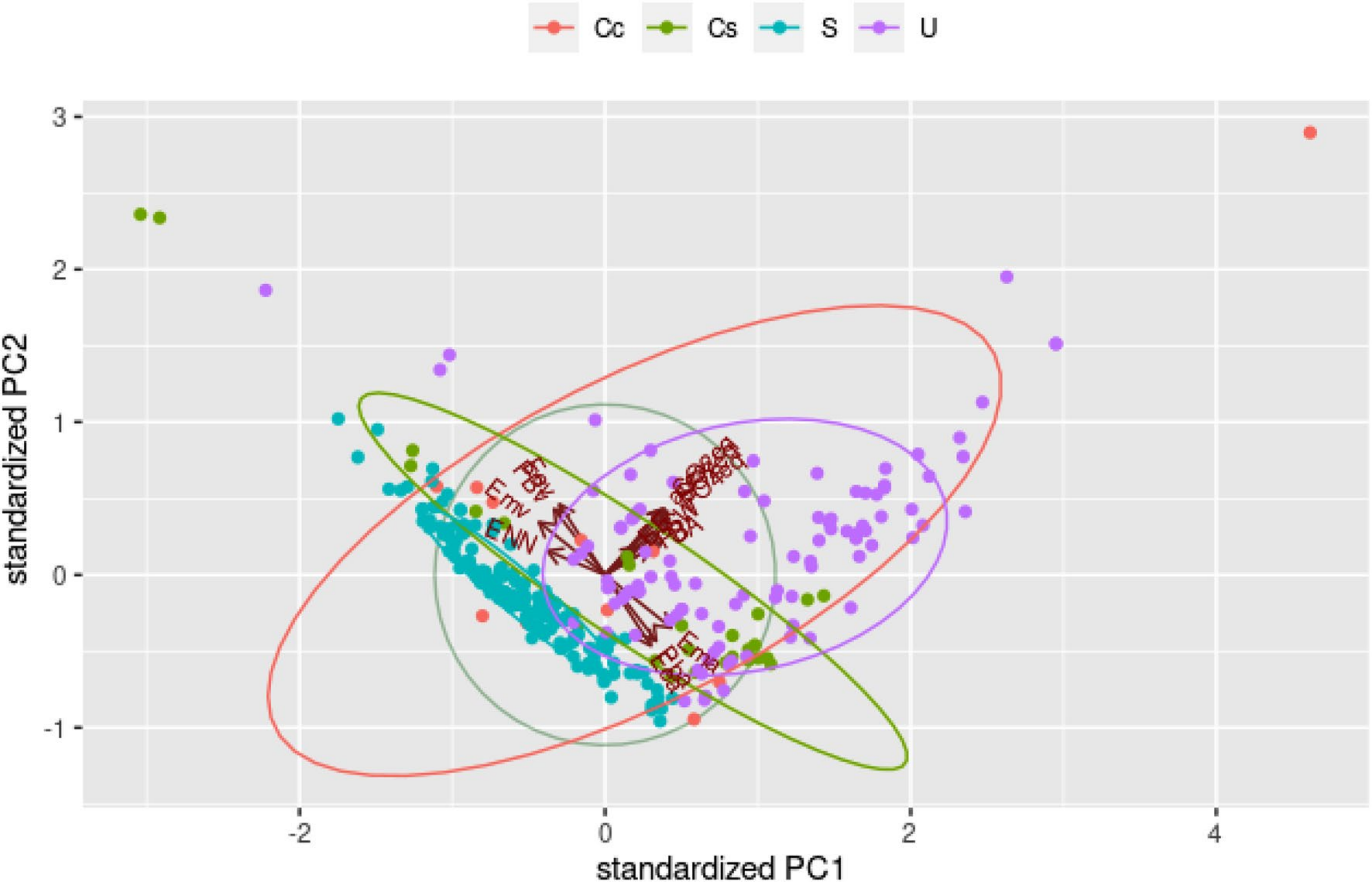
Biplot of standardized first (PC1) and second principal components (PC2) with observations of diets for cats. *Cc *homemade diets, *Cs* website homemade diets, *S* dry diets, *U* wet diets, *ENN* nitrogen-free extract, *PBv* protein from vegetable sources, *Emv* metabolizable energy from vegetable sources, *Eev*  fat from vegetable sources, *CO2eq* carbon dioxide equivalent, *area* land use, *PO4eq* phosphate equivalent, *SW* stress-weighted water usage, *SO2eq* sulphur oxide equivalent, *UA* freshwater withdrawals, *PB* protein content, *EE* fat content, *Ema* metabolizable energy from animal sources, *Pba* protein from animal sources, *Eea* fat from animal sources.

Similar to dogs, the wet diets for cats correlated to an increased environmental impact, as the diet observations follow the same direction as the vectors of the variables for environmental impact (Fig. 7).

## Discussion

In the present study, extensive research regarding the composition of different types of pet food was performed to estimate the environmental impact of diets for dogs and cats in Brazil. This approach allowed to estimate the impact of different variables of environmental impact, which revealed that wet diets positively correlated to higher environmental impact than dry or homemade diets.

If a 10 kg dog with an average caloric intake of 534 kcal per day^15^ is considered, we can estimate the yearly consumption of calories and, therefore, can estimate the annual environmental impact. If we consider the results of the present study, the median of CO_2_eq of a dry diet per 1000 kcal is 4.25 kg and a wet diet of 33.56 kg. This average dog would be responsible for 828.37 kg of CO_2_eq per year if consuming dry diets or 6,541 kg of CO_2_eq per year if consuming wet diets. This is consistent with 12.4 to 97.8% of the emission of a Brazilian citizen, which is 6.69 tCO_2_eq per year^16^. If we extrapolate this emission to the canine population in Brazil, of 52.2 million (*2*), the total emission would be between 0.04 and 0.34 Gt CO_2_eq per year, which would represent from 2.9 to 24.6% of the total estimated emission of 1.38 Gt for Brazil^16^. These results bring to light the importance of the role of pet food in the discussion of sustainability since its impact can be extensive.

In the present study, it was observed that dry pet foods caused lower environmental impact because the environmental impact variables studied (all variables for dogs and CO_2_eq, land use, and PO_4_^3−^ eq for cats) were lower per 1000 kcal. However, the number of veterinarians, breeders and pet owners interested in homemade or home-prepared diets seems to be increasing^17–20^. Our data showed, however, that this type of diet is related to a higher environmental impact than conventional dry diets. Wet diets, whilst indicated as a strategy to increase palatability and water consumption by cat and dogs with a higher risk of developing urolithiasis^21,22^, were the ones that had the highest environmental impact.

Many factors can influence the sustainability of food, including ingredient choice, ingredient composition, digestibility, and percentage of ingredient inclusion. Sometimes the ingredient choice is made taking into consideration consumer demand instead of only nutritional composition, which can lead to ingredients that compete directly with human diets. Furthermore, diets are sometimes formulated to contain an excess of nutrients. These factors represent a challenge to optimize the sustainability of pet food^5^.

In the subject of sustainability of the pet food system, animal protein is almost always in the spotlight. Animal proteins usually have higher CO_2_eq emissions than proteins from vegetables. For example, the production of 100 g of pea protein is responsible for the emission of 0.4 kg CO_2_eq, while the production of the same amount of protein from beef is responsible for 35.0 kg CO_2_eq, almost 90 times more^7^. Even when comparing the pea farm with the highest carbon footprint (0.8 kg CO_2_eq/100 g protein) with the lowest farm of beef or chicken production (9.0 and 2.4 kg CO_2_eq/100 g protein, respectively), there is an important difference between plant- and animal-based proteins. Human plant-based diets or diets with more plant-based protein require less energy, less freshwater, and less land use when compared to diets with more ingredients of animal sources^23^. Dogs and cats, however, have different nutritional requirements and are considered carnivores^15,24^ and, therefore, vegan diets could lead to risks for these species^25^. Specifically, about proteins requirements, synthetic amino acids can be added to pet foods as a way to correct possible nutritional imbalances^24^, but environmental impact of this addition was not evaluated.

The present study observed that most of the proteins of the diets were from animal origin. Despite the intake of vegetable proteins having a lower impact on the environment, in the case that animal protein needs to be included the choice for production with lower impact is important. According to the Food and Agricultural Organization (FAO), 61% of pork production, 81% of chicken production, and 86% of egg production use intensive farming methods, which can reduce considerably the impact on the environment, especially regarding land use and CO_2_eq emission^26^. In this case, products of extensive farming, especially those from pastures from deforestation as occurs in most developing countries, can represent a higher impact, and therefore should be avoided^27^. However, other studies showed that pasture development minimizes the environmental impact of extensive farming due to pasture consumes part of the GHGs produced by animal production, and different pasture management strategies can be effective alternative for sustainable animal protein production^28,29^*.*

Several ingredients used in pet food are considered by-products, and this could be considered as a factor that reduces the impact of these foods^5,13^. According to the Brazilian Association of Animal Rendering^30^, ingredients produced by rendering are named *non-edible products of animal origin*, which include meals, fats, and blood derivates. According to a report from this institution, approximately 38% of beef, 20% of pork, and 19% of chicken is viscera or blood that is not used for human consumption^31^. Of all the by-products produced in Brazil, 12.8% are used in the pet food segment, and the rest is used for animal production, biodiesel, hygiene, and cleaning, among other uses^32^. There is no information on how much of these by-products are turned into meals and fats and how much is used fresh, or even if the fresh offal is not considered in this calculation of rendering potential. The argument that consuming by-product is more sustainable and therefore should not be considered when estimating the environmental impact of pet food, however, can be partially true. Fresh offal can sometimes compete with human markets and there may not be sufficient by-products from the industry of human food to feed the increasing population of pets, which means that animal production could need an increase due to pet food demand^32^. In the present study, all types of diets contained by-products such as offal or meat meals, although dry and wet diets presented by-products more often than homemade diets.

The pet food industry should have diets that are accepted by the owners and at the same time be nutritionally balanced and palatable for the pets. There is no single strategy for improving sustainability that applies to all manufacturers since regional demand and socioeconomic development must be taken into consideration^5^. Suggestions to promote more sustainable pet food include the use of alternative protein sources. As protein-rich ingredients can be one of the main sources of environmental impact, the choice of protein type is very important, not only between vegetable or animal sources but among different species, such as beef, pork, chicken, or fish. The different sources of protein have different impacts regarding sustainability^7^ and, therefore, a change of inclusion or ingredient should be considered depending on the nutrient requirement and diet composition as a whole. Furthermore, the inclusion of alternative ingredients, such as insects, could improve the sustainability of a diet. The estimated CO_2_eq emission per 100 g of protein from mealworms (*Tenebrio molitor*) is 14 kg, and the use is approximately 18 m^2^, which can be up to 14 times less than chicken, pork, or beef production^33^.

Another possibility of providing a more sustainable diet for pets is to avoid providing nutrients in excess. Our data showed that diet with higher NFE caused lower environmental impact. The daily recommended intake of protein according to FEDIAF^15^ per 1000 kcal is 52.1 g for inactive dogs and 83.3 g for inactive cats, and the daily recommendation for fat intake is 13.75 g for inactive dogs and 22.5 g for inactive cats. All types of diets included in the present study provided more protein and fat than recommended for dogs and cats. Amino acids provided by the extra protein are not stored in the organism and can either be utilized as an energy source or be excreted. Fatty acids provided by excessive fat, on the other hand, are utilized as an energy source or stored as fat deposits, which can lead to obesity^24^. This excessive intake of nutrients can be seen as a potential waste of resources from a sustainable point of view^5^. However, sometimes higher protein and fat contents in diets can be used to enhance the acceptance of diets by pets, and a balance should be thought between excessive nutrients and palatability of the diet.

## Materials and methods

### Diet selection

To estimate the environmental impact, data was collected from different types of pet food for healthy adults. All pet foods were categorized as dry (extruded pet food with 12% or less moisture), wet (canned or pouch), and homemade diets (produced using the same ingredients as man food). Homemade pet foods were subcategorized as "commercial homemade" (produced and sold by pet food companies) or "website homemade" (recipes recommended by websites to be cooked at home by owners). To estimate the environmental impact of commercial pet foods, all commercial dry and wet diets found on the websites of the three major retailers of the pet food sector in Brazil were selected. Commercial homemade diets were selected after a search using the Google search tool using the Portuguese terms for “buy” and "homemade diet", followed by the terms “dog”, “canine”, “cat” or “feline”. Website recipes published in Portuguese were selected using the Google search tool, and search terms were “homemade diet recipe” and “homemade food recipe” followed by the terms “dog” and “cat”. For both commercial and website homemade diets, the results obtained up to the 10th page of the search tool for each term were considered.

All diets included were advertised as complete and balanced for healthy adults, and exclusion criteria were diets for puppies or kittens, senior diets, therapeutic diets, and treats. Website homemade diet recipes were excluded if the quantity of one or more ingredients was not specified and the same recipes on different websites were also not included.

### Ingredient inclusion percentage

Information regarding the ingredients (except premixes, additives, and preservatives) and guaranteed analysis from labels of all commercial diets were collected. For the recipes of homemade diets acquired from websites, the ingredients and their amounts were considered as described by the website’s authors.

The ingredient inclusion percentages for each commercial diet were estimated using a diet formulation software^34^, aiming at the dry matter macronutrient concentration. The guaranteed analysis information of macronutrients (crude protein, crude fat, crude fiber, and ash) was converted to a dry matter basis according to the moisture declared on the label. This information was then inserted into the nutrient composition part of each diet in the software.

For nutrients with minimum guaranteed levels (crude fat and crude protein), values for maximum inclusion in the software were considered as up to 10% of the minimum value. For nutrients with maximum guaranteed levels (crude fiber and ash), only maximum levels were inserted in the software.

The ingredient database for commercial wet and dry diets was obtained preferably from the Brazilian Association of the Pet Food Industry (ABINPET)^35^, but when not described in this publication, other sources were used^36,37^. For the homemade diets (commercial and website), the ingredient database was obtained from the USDA’s FoodData Central^37^ or, when not presented at FoodData Central, the Brazilian Table of Food Composition (TACO)^38^ was used.

After the percentages of inclusion of ingredients were estimated in a dry matter basis, they were converted to percentage of inclusion in original matter basis (as fed), considering the ingredients’ moisture^35–37^.

For the website homemade diet recipes, the amount in original matter basis was already stated, and inclusion percentage was calculated according with total amount of the recipe and the amount of each ingredient.

### Macronutrient profile

The quantities of protein, fat and nitrogen-free extract (NFE) of the diets were calculated according to label information provided by the manufacturers. The information regarding the metabolizable energy and the minimum amounts of crude protein and crude fat according to the guaranteed analysis information were obtained, and with this information the amount of nutrient per 1000 kcal of the diet was estimated for the dry, wet, and commercial homemade diets. For these three types of diets, the NFE was calculated according to the NRC^24^ equation:

$$ \% {\text{ NFE}}\, = \,{1}00{-}(\% {\text{ crude\, protein}}\, + \,\% {\text{ crude\, fat}}\, + \,\% {\text{ crude \, fiber}}\, + \,\% {\text{ ash}}\, + \,\% {\text{ moisture}}). $$

For the website homemade diets, the information was obtained by the composition of the recipe, as they did not contain labels. According with the recipe, the metabolizable energy, protein, fat, and NFE were estimated based on the composition of nutrients^37^.

The metabolizable energy of each diet as informed by the manufacturers on the labels was considered for commercial dry, wet and homemade diets. The Atwater method was used to calculate the energy of website homemade diets^24^, considering 4 kcal per gram of protein and NFE and 9 kcal per gram of fat^39^.

### Nutrient and energy source estimate

To better understand the source of nutrients of diets for dogs and cats, the percentage of protein, fat and metabolizable energy provided by vegetable or animal ingredients was calculated for each diet. The percentage was calculated according to the contribution of the nutrient provided by each ingredient in the diet, and if this ingredient was of animal or vegetable origin. For the calculation of the energy source percentage, the energy provided by each ingredient type was considered.

### Environmental impact estimate

The environmental impact variables evaluated were greenhouse gas (GHG) emission (as carbon dioxide equivalent emission—CO_2_eq), land use, acidifying emission (as sulphur dioxide equivalent emission—SO_2_eq), eutrophying emissions (as phosphate equivalent emissions—PO_4_^3−^eq), freshwater withdrawals, and stress-weighted water use per 1000 kcal of diet, according with the metabolizable energy of the diet and the percentage of inclusion of each ingredient in the diet, as the equation below:

$$Impact\, variable\, per\, 1000 \,\mathrm{kcal}=\frac{Amount \,of\, ingredient/1000 \,\mathrm{kcal} \,of \,diet \,\times \,Variable/1000 \,\mathrm{kcal} \,of \,ingredient}{1000\, \mathrm{kcal}}$$

To obtain these results, the diet composition was first converted from a dry matter basis to a 1000 kcal basis using the following equation, applied to all ingrediets present in the diet:

$$Amount\, of \,ingredient\, per\, 1000 \,\mathrm{kcal}= \frac{Amount \,of\, ingredient\, per\, kg\, of \,diet\, \times \,1000}{Metabolizable\, energy\, of \,the \,diet\, (\mathrm{kcal}/\mathrm{kg})}$$

The comparison per 1000 kcal was used to put all diets on a basis of dietary intake, as a dog or cat requires the same energy intake regardless of the diet chosen and is a reliable unit to compare dietary composition and nutrient intake.

The data used to estimate the variables of environmental impact was based on the data from Poore and Nemecek^7^ for nutrition functional units as 1000 kcal. When data was provided per 100 g protein or per kg of product, it was converted to 1000 kcal based on data from ABINPET^35^, Butolo^36^, TACO^38^, and USDA^37^ (Table S4). The ingredients were classified in one of the 43 groups listed by Poore and Nemecek^7^, for example, all types of beef meat were calculated as bovine meat.

Furthermore, the relationship between the dietary nutrient composition and the variables that were used to evaluate the environmental impact was assessed.

### Statistical analysis

The statistical analysis was performed using R Core Team^40^. Adherence to normality was tested with the Shapiro–Wilk test, and as only the variables crude protein concentration and NFE of website homemade diets, and SO_2_eq of dry diets were considered to adhere to normality, non-parametric tests were performed. For the analysis of macronutrient profile and the estimated environmental impact, the Kruskal–Wallis test was used to compare variables. When at least one median was considered different, multiple comparisons between groups were performed. The comparison between energy provided by ingredients of vegetable and animal origin was performed with the Wilcoxon test, considering the variables as two dependent samples. Values of p < 0.05 were considered significant.

The principal component analysis (PCA) was used to evaluate the relation between the diet characteristics and the variables of environmental impact. As the units of the variables were different, they were scaled considering mean = 0 and variance = 1. The first (PC1) and second principal components (PC2) were responsible for 68.2% of the data variance for dogs (Fig. S3). For cats, PC1 and PC2 are responsible for 71.1% of the data variance (Fig. S4).

## Supplementary Information

Supplementary Information.

## Data Availability

The datasets generated during and/or analyzed during the current study are available from the corresponding author on reasonable request.
