# Supplementary material for: Environmental impact of diets for dogs and cats
Source: Sci Rep. 2022 Nov 17;12:18510. doi: 10.1038/s41598-022-22631-0 (PMC9672041; doi:10.1038/s41598-022-22631-0)
Supplement: Supplementary file 1 — Supplementary Information. [file 41598_2022_22631_MOESM1_ESM.docx]

Supplementary Materials for

Environmental impact of diets for dogs and cats

Vivian Pedrinelli, Fabio A. Teixeira, Mariana R. Queiroz, Marcio A. Brunetto

**Table S1.** Median values for the percentage of metabolizable energy, crude protein, and crude fat according to the source (animal or vegetable) of diets for dogs included in the study.

|  | Metabolizable energy (%) | | *p* | Protein (%) | | *p* | Fat (%) | | *p* |
| --- | --- | --- | --- | --- | --- | --- | --- | --- | --- |
|  | Animal source | Vegetable source |  | Animal source | Vegetable source |  | Animal source | Vegetable source |  |
| Dry diets | 45.42 | 54.58 | <0.01 | 72.71 | 27.29 | <0.01 | 86.84 | 13.16 | <0.01 |
| Wet diets | 89.27 | 10.73 | <0.01 | 94.23 | 5.77 | <0.01 | 99.13 | 0.87 | <0.01 |
| Commercial homemade diets | 47.07 | 52.93 | 0.1 | 84.57 | 15.43 | <0.01 | 60.50 | 39.50 | <0.01 |
| Website homemade diets | 46.91 | 53.09 | 0.09 | 84.25 | 15.74 | <0.01 | 69.64 | 30.06 | <0.01 |

**Table S2.** Median values for the percentage of metabolizable energy, crude protein, and crude fat according to the source (animal or vegetable) of diets for cats included in the study.

|  | Metabolizable energy (%) | | *p* | Protein (%) | | *p* | Fat (%) | | *p* |
| --- | --- | --- | --- | --- | --- | --- | --- | --- | --- |
|  | Animal source | Vegetable source |  | Animal source | Vegetable source |  | Animal source | Vegetable source |  |
| Dry diets | 51.31 | 48.69 | <0.01 | 76.02 | 23.98 | <0.01 | 88.72 | 11.28 | <0.01 |
| Wet diets | 83.63 | 16.37 | <0.01 | 88.31 | 11.69 | <0.01 | 98.02 | 1.98 | <0.01 |
| Commercial homemade diets | 69.63 | 30.37 | <0.01 | 90.98 | 9.02 | <0.01 | 78.83 | 21.17 | 0.03 |
| Website homemade diets | 87.34 | 12.66 | <0.01 | 95.46 | 4.54 | <0.01 | 97.43 | 2.57 | <0.01 |


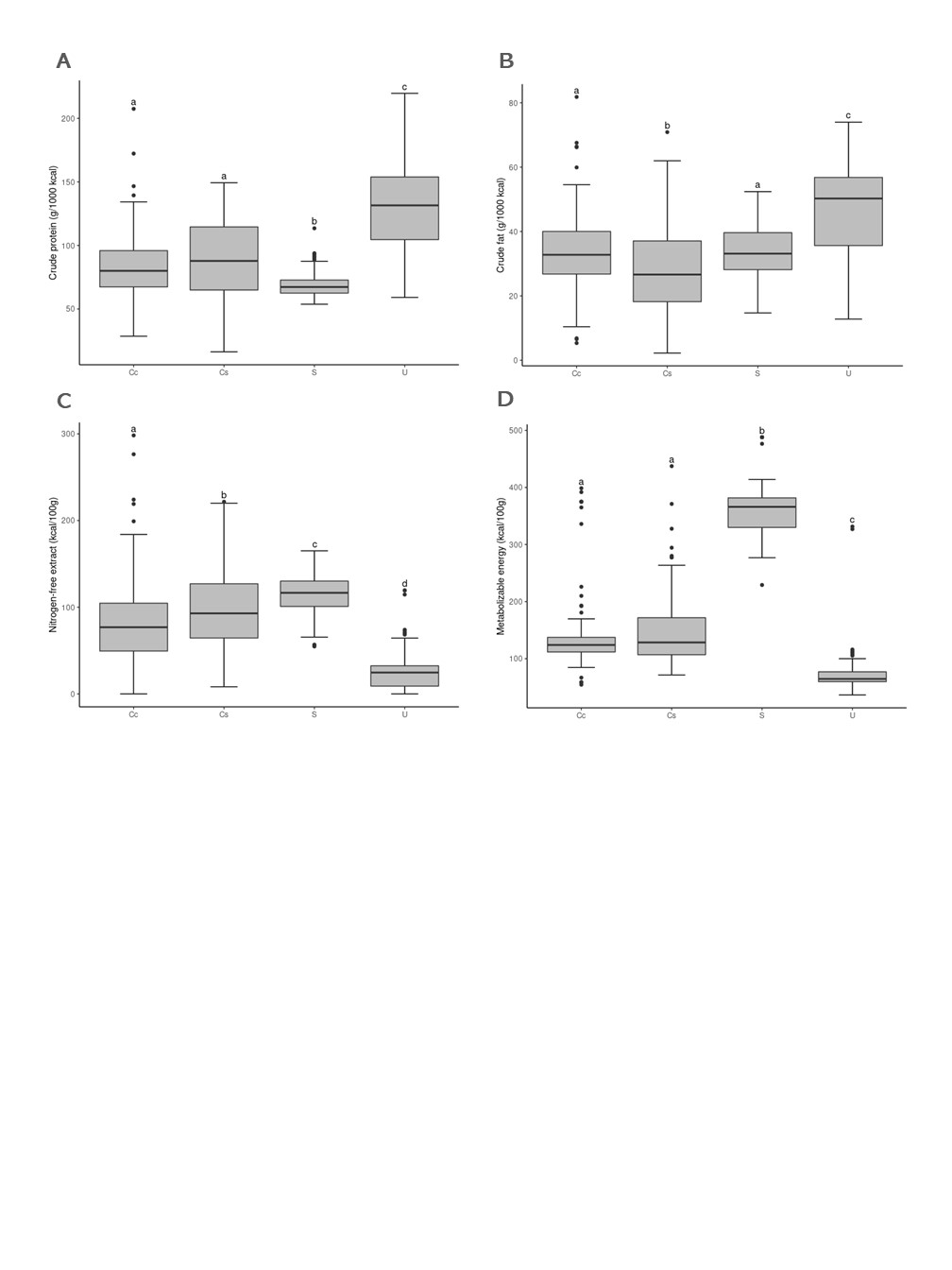


Fig. S1.

Profile of metabolizable energy, crude protein, crude fat and nitrogen-free extract of each category of diet for dogs: crude protein (A), crude fat (B), nitrogen-free extract (C) and metabolizable energy in a dry matter basis (D). Diet category: Cc = homemade diets; Cs = website homemade diets; S = dry diets; and U = wet diets. Plots of the same variable that have different letters differed (p<0.05) according to the multiple comparison test between groups.


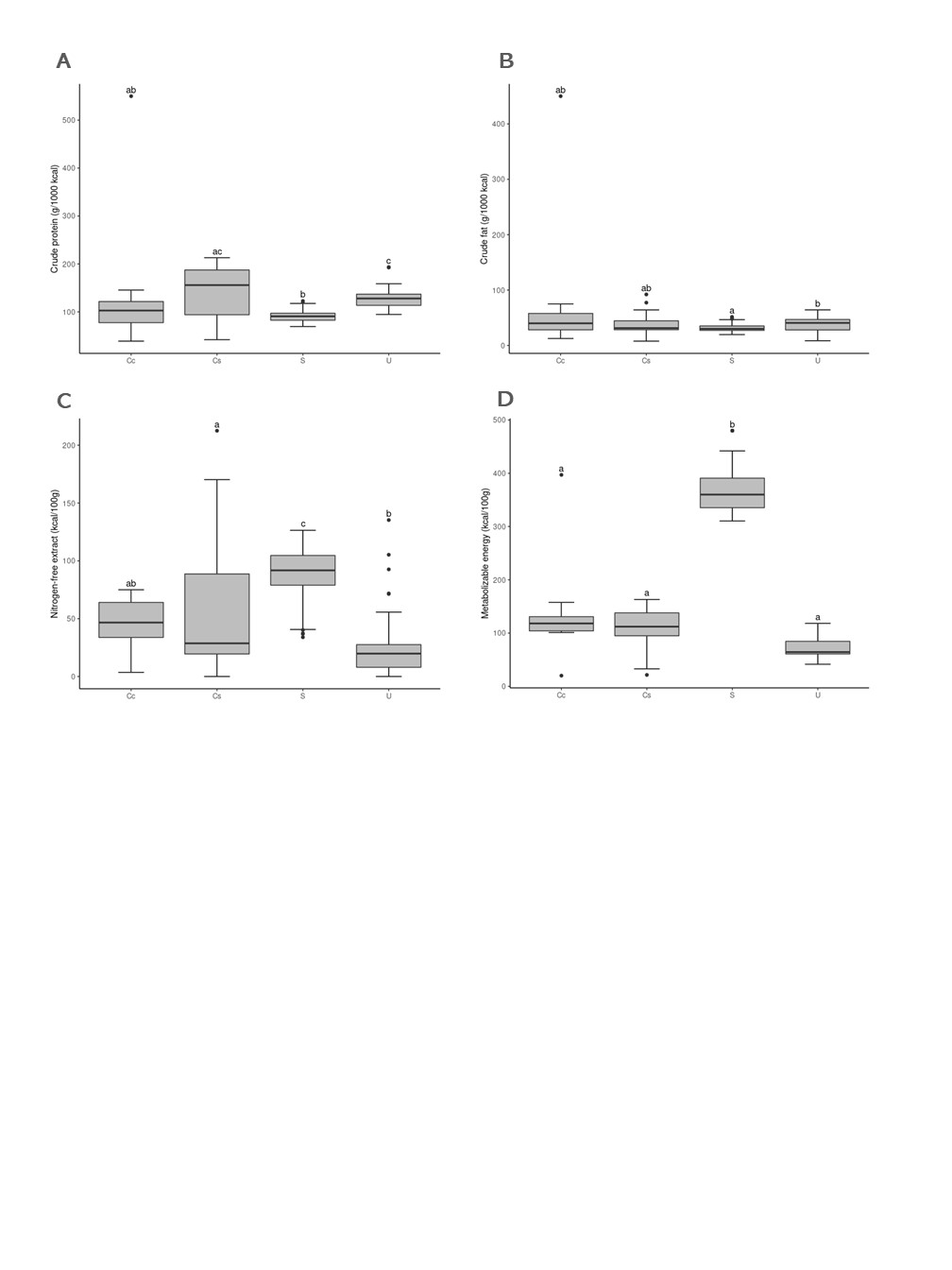


Fig. S2.

Profile of metabolizable energy, crude protein, crude fat and nitrogen-free extract of each category of diet for cats: crude protein (A), crude fat (B), nitrogen-free extract (C) and metabolizable energy in a dry matter basis (D). Diet category: Cc = homemade diets; Cs = website homemade diets; S = dry diets; and U = wet diets. Plots of the same variable that have different letters differed (p<0.05) according to the multiple comparison test between groups.


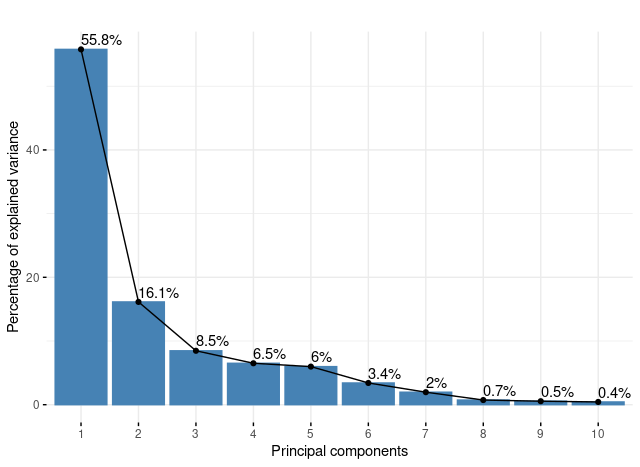


Fig. S3.

Explained variance percentage of principal components of diets for dogs for the principal component analysis (PCA).


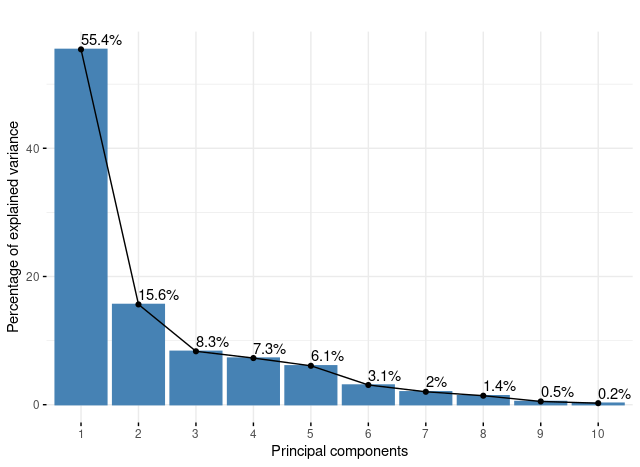


Fig. S4.

Explained variance percentage of principal components of diets for cats for the principal component analysis (PCA).

Table S3. List of ingredients and inclusion in the diets evaluated in the study.

|  | Dogs | | Cats | | Total  (n=938) |
| --- | --- | --- | --- | --- | --- |
| Ingredient | Commercial and website homemade diets (n=221) | Dry and wet diets  (n=397) | Commercial and website homemade diets (n=37) | Dry and wet diets (n=283) |  |
| Apple pomace, unspecified | 0 | 11 | 0 | 23 | 34 |
| Apple, raw | 2 | 0 | 0 | 0 | 2 |
| Arracacha, cooked | 21 | 0 | 3 | 0 | 24 |
| Artichoke, unspecified | 0 | 1 | 0 | 1 | 2 |
| Bacon, cooked | 2 | 0 | 0 | 0 | 2 |
| Banana, raw | 1 | 0 | 0 | 0 | 1 |
| Barley, cooked | 4 | 0 | 0 | 0 | 4 |
| Barley, unspecified | 0 | 25 | 0 | 13 | 38 |
| Beef heart, cooked | 34 | 0 | 8 | 0 | 42 |
| Beef heart, raw | 2 | 0 | 4 | 0 | 6 |
| Beef kidney, cooked | 5 | 0 | 3 | 0 | 8 |
| Beef kidney, raw | 1 | 0 | 2 | 0 | 3 |
| Beef liver, cooked | 54 | 0 | 9 | 0 | 63 |
| Beef liver, raw | 3 | 0 | 2 | 0 | 5 |
| Beef lung, cooked | 0 | 0 | 1 | 0 | 1 |
| Beef lung, raw | 0 | 0 | 1 | 0 | 1 |
| Beef meal, unspecified | 0 | 15 | 0 | 5 | 20 |
| Beef meat, cooked | 55 | 0 | 11 | 0 | 66 |
| Beef meat, unspecified | 0 | 19 | 0 | 20 | 39 |
| Beef offal, unspecified | 2 | 18 | 2 | 21 | 43 |
| Beef shoulder clod, cooked | 20 | 0 | 4 | 0 | 24 |
| Beef spleen, cooked | 1 | 0 | 0 | 0 | 1 |
| Beef spleen, raw | 1 | 0 | 0 | 0 | 1 |
| Beef tallow, unspecified | 6 | 34 | 0 | 42 | 82 |
| Beef tongue, cooked | 4 | 0 | 2 | 0 | 6 |
| Beef tongue, raw | 0 | 0 | 1 | 0 | 1 |
| Beet pulp, unspecified | 0 | 196 | 0 | 120 | 316 |
| Beets, cooked | 14 | 0 | 3 | 0 | 17 |
| Bell pepper, cooked | 2 | 0 | 3 | 0 | 5 |
| Blueberry, unspecified | 8 | 3 | 0 | 2 | 13 |
| Boar meat, unspecified | 1 | 1 | 0 | 0 | 2 |
| Borage seed oil, unspecified | 5 | 0 | 0 | 0 | 5 |
| Brazilian nut, raw | 1 | 0 | 0 | 0 | 1 |
| Brewer's rice, unspecified | 0 | 17 | 0 | 8 | 25 |
| Brewer's yeast, unspecified | 48 | 134 | 8 | 119 | 309 |
| Broccoli leaves, cooked | 2 | 0 | 0 | 0 | 2 |
| Broccoli, cooked | 31 | 0 | 6 | 0 | 37 |
| Broken bean, unspecified | 7 | 4 | 2 | 0 | 13 |
| Broken rice, unspecified | 0 | 218 | 0 | 124 | 342 |
| Brown rice, cooked | 54 | 0 | 5 | 0 | 59 |
| Butter, unspecified | 1 | 0 | 0 | 0 | 1 |
| Cabbage, cooked | 1 | 0 | 2 | 0 | 3 |
| Canola oil, unspecified | 15 | 0 | 3 | 0 | 18 |
| Carrot, cooked | 115 | 0 | 19 | 0 | 134 |
| Carrot, unspecified | 0 | 11 | 0 | 29 | 40 |
| Cauliflower, cooked | 7 | 0 | 0 | 0 | 7 |
| Celery, raw | 3 | 0 | 3 | 0 | 6 |
| Cellulose, unspecified | 8 | 66 | 0 | 55 | 129 |
| Chard, cooked | 1 | 0 | 0 | 0 | 1 |
| Chayote, cooked | 70 | 0 | 3 | 0 | 73 |
| Cheese curds, unspecified | 6 | 0 | 1 | 0 | 7 |
| Chia seeds, unspecified | 8 | 2 | 0 | 0 | 10 |
| Chicken breast, cooked | 54 | 0 | 8 | 0 | 62 |
| Chicken breast, raw | 1 | 0 | 1 | 0 | 2 |
| Chicken breast, unspecified | 0 | 2 | 0 | 0 | 2 |
| Chicken drumsticks, cooked | 8 | 0 | 1 | 0 | 9 |
| Chicken gizzard, cooked | 14 | 0 | 3 | 0 | 17 |
| Chicken gizzard, raw | 1 | 0 | 1 | 0 | 2 |
| Chicken liver hydrolysate, unspecified | 0 | 67 | 0 | 41 | 108 |
| Chicken liver, cooked | 27 | 0 | 0 | 0 | 27 |
| Chicken meat, cooked | 7 | 8 | 4 | 26 | 45 |
| Chicken meat, raw | 1 | 0 | 0 | 0 | 1 |
| Chicken neck, raw | 0 | 0 | 2 | 0 | 2 |
| Chicken liver hydrolysate, unspecified | 0 | 0 | 1 | 0 | 1 |
| Chickpea, cooked | 3 | 0 | 0 | 0 | 3 |
| Chicory, cooked | 1 | 0 | 0 | 0 | 1 |
| Chinese cabbage, cooked | 4 | 0 | 1 | 0 | 5 |
| Coconut oil, unspecified | 31 | 0 | 0 | 0 | 31 |
| Coconut pulp, unspecified | 0 | 4 | 0 | 2 | 6 |
| Coconut, raw | 2 | 0 | 1 | 0 | 3 |
| Cod live roil, unspecified | 3 | 0 | 0 | 0 | 3 |
| Collagen, unspecified | 2 | 3 | 0 | 3 | 8 |
| Collards, cooked | 27 | 0 | 4 | 0 | 31 |
| Corn germ, unspecified | 0 | 10 | 0 | 0 | 10 |
| Corn gluten meal 21, unspecified | 0 | 37 | 0 | 32 | 69 |
| Corn gluten meal 60, unspecified | 0 | 83 | 0 | 106 | 189 |
| Corn oil, unspecified | 5 | 0 | 1 | 0 | 6 |
| Cornmeal, unspecified | 3 | 0 | 0 | 0 | 3 |
| Defatted rice bran, unspecified | 0 | 43 | 0 | 0 | 43 |
| Dextrose, unspecified | 1 | 0 | 0 | 0 | 1 |
| Duck meat, cooked | 2 | 0 | 1 | 0 | 3 |
| Duck meat, unspecified | 0 | 2 | 0 | 1 | 3 |
| Egg powder, unspecified | 0 | 113 | 0 | 61 | 174 |
| Egg, cooked | 51 | 0 | 13 | 0 | 64 |
| Eggplant, cooked | 7 | 0 | 2 | 0 | 9 |
| Escarole, cooked | 4 | 0 | 0 | 0 | 4 |
| Farro, cooked | 0 | 2 | 0 | 0 | 2 |
| Fish meal, unspecified | 0 | 42 | 0 | 44 | 86 |
| Fish oil, unspecified | 15 | 7 | 0 | 10 | 32 |
| Fish protein hydrolysate, unspecified | 0 | 3 | 0 | 3 | 6 |
| Flaxseed oil, unspecified | 22 | 0 | 0 | 0 | 22 |
| Flaxseed, unspecified | 38 | 121 | 0 | 46 | 205 |
| Garlic, raw | 8 | 0 | 0 | 0 | 8 |
| Green beans, cooked | 25 | 0 | 2 | 0 | 27 |
| Hake, cooked | 2 | 0 | 0 | 0 | 2 |
| Hemoglobin powder, unspecified | 0 | 7 | 0 | 13 | 20 |
| Herring, unspecified | 1 | 9 | 0 | 14 | 24 |
| Lamb meal, unspecified | 0 | 4 | 0 | 0 | 4 |
| Lamb meat and bone meal, unspecified | 0 | 24 | 0 | 8 | 32 |
| Lamb meat, cooked | 6 | 0 | 1 | 0 | 7 |
| Lamb meat, unspecified | 0 | 20 | 0 | 9 | 29 |
| Lamb offal, unspecified | 1 | 5 | 0 | 2 | 8 |
| Leek, cooked | 2 | 0 | 0 | 0 | 2 |
| Lentil, cooked | 15 | 0 | 3 | 0 | 18 |
| Lignocellulose, unspecified | 0 | 26 | 0 | 0 | 26 |
| Meat and bone meal, unspecified | 0 | 78 | 0 | 40 | 118 |
| Mechanically separated beef meat, unspecified | 0 | 7 | 0 | 0 | 7 |
| Mechanically separated chicken meat, unspecified | 3 | 105 | 3 | 85 | 196 |
| Milk cream, unspecified | 1 | 0 | 1 | 0 | 2 |
| Millet, cooked | 1 | 0 | 0 | 0 | 1 |
| Mozzarella, unspecified | 5 | 0 | 0 | 0 | 5 |
| Oat bran, raw | 8 | 0 | 0 | 0 | 8 |
| Oat flour, unspecified | 0 | 7 | 0 | 7 | 14 |
| Okra, cooked | 5 | 0 | 2 | 0 | 7 |
| Olive oil, unspecified | 54 | 0 | 1 | 0 | 55 |
| Onion, raw | 1 | 0 | 0 | 0 | 1 |
| Ostrich meat, unspecified | 1 | 0 | 1 | 0 | 2 |
| Papaya, raw | 1 | 0 | 1 | 0 | 2 |
| Parboiled rice, cooked | 36 | 0 | 0 | 0 | 36 |
| Pea flour, unspecified | 0 | 19 | 0 | 5 | 24 |
| Pea hull, unspecified | 0 | 38 | 0 | 21 | 59 |
| Pea, cooked | 27 | 0 | 5 | 0 | 32 |
| Pea, unspecified | 0 | 7 | 0 | 9 | 16 |
| Peanut butter, unspecified | 1 | 0 | 0 | 0 | 1 |
| Plasma powder, unspecified | 0 | 39 | 3 | 57 | 99 |
| Pork ham, cooked | 8 | 0 | 1 | 0 | 9 |
| Pork ham, raw | 1 | 0 | 0 | 0 | 1 |
| Pork heart, cooked | 1 | 0 | 0 | 0 | 1 |
| Pork kidney, cooked | 1 | 0 | 0 | 0 | 1 |
| Pork liver, unspecified | 0 | 27 | 0 | 31 | 58 |
| Pork loin, cooked | 12 | 0 | 1 | 0 | 13 |
| Pork lung, cooked | 1 | 0 | 0 | 0 | 1 |
| Pork meat and bone meal, unspecified | 0 | 16 | 0 | 2 | 18 |
| Pork offal, unspecified | 0 | 33 | 0 | 38 | 71 |
| Pork protein isolate, unspecified | 0 | 104 | 0 | 79 | 183 |
| Pork tallow, unspecified | 11 | 28 | 0 | 9 | 48 |
| Pork tenderloin, cooked | 4 | 0 | 1 | 0 | 5 |
| Pork tenderloin, raw | 0 | 0 | 1 | 0 | 1 |
| Potato starch, unspecified | 1 | 53 | 0 | 56 | 110 |
| Potato, cooked | 20 | 0 | 1 | 0 | 21 |
| Poultry by-product meal, unspecified | 4 | 297 | 0 | 187 | 488 |
| Poultry fat, unspecified | 0 | 300 | 0 | 178 | 478 |
| Poultry liver, unspecified | 0 | 29 | 0 | 65 | 94 |
| Poultry offal, unspecified | 5 | 40 | 3 | 56 | 104 |
| Powdered milk, unspecified | 4 | 0 | 0 | 0 | 4 |
| Pumpkin seed, toasted | 5 | 0 | 0 | 0 | 5 |
| Quick oats, raw | 35 | 0 | 3 | 0 | 38 |
| Quinoa, cooked | 14 | 0 | 0 | 0 | 14 |
| Quinoa, unspecified | 0 | 8 | 0 | 8 | 16 |
| Rabbit meat, raw | 0 | 0 | 1 | 0 | 1 |
| Rabbit meat, unspecified | 1 | 0 | 1 | 0 | 2 |
| Rice flour, unspecified | 1 | 4 | 0 | 1 | 6 |
| Rice noodle, cooked | 11 | 0 | 1 | 0 | 12 |
| Ricotta, unspecified | 1 | 0 | 0 | 0 | 1 |
| Salmon meal, unspecified | 0 | 17 | 0 | 26 | 43 |
| Salmon pulp, unspecified | 0 | 8 | 0 | 23 | 31 |
| Salmon, cooked | 1 | 0 | 0 | 0 | 1 |
| Sardine pulp, unspecified | 0 | 0 | 0 | 6 | 6 |
| Sardine, cooked | 14 | 0 | 8 | 0 | 22 |
| Sardine, unspecified | 0 | 0 | 0 | 15 | 15 |
| Sesame seed oil, unspecified | 1 | 0 | 0 | 0 | 1 |
| Shrimp meal, unspecified | 0 | 0 | 0 | 3 | 3 |
| Shrimp, cooked | 1 | 1 | 0 | 3 | 5 |
| Sorghum, unspecified | 0 | 47 | 0 | 23 | 70 |
| Soy bran, unspecified | 0 | 67 | 0 | 46 | 113 |
| Soy fiber, unspecified | 0 | 15 | 0 | 16 | 31 |
| Soy flour, unspecified | 7 | 0 | 0 | 0 | 7 |
| Soy hull, unspecified | 0 | 9 | 0 | 12 | 21 |
| Soy protein concentrate, unspecified | 0 | 18 | 0 | 38 | 56 |
| Soy protein hydrolysate, unspecified | 0 | 3 | 0 | 0 | 3 |
| Soy protein isolate, unspecified | 0 | 15 | 0 | 15 | 30 |
| Soy, cooked | 1 | 0 | 0 | 0 | 1 |
| Soybean oil, unspecified | 23 | 52 | 3 | 24 | 102 |
| Spaghetti, cooked | 2 | 0 | 0 | 0 | 2 |
| Spinach, cooked | 14 | 0 | 2 | 0 | 16 |
| Squash, cooked | 69 | 0 | 10 | 0 | 79 |
| Squash, unspecified | 0 | 7 | 0 | 8 | 15 |
| Squid meal, unspecified | 0 | 0 | 0 | 1 | 1 |
| Sugarcane fiber, unspecified | 5 | 0 | 0 | 0 | 5 |
| Sunflower oil, unspecified | 46 | 0 | 5 | 0 | 51 |
| Sunflower seed, toasted | 3 | 0 | 0 | 0 | 3 |
| Sweet potato flour, unspecified | 0 | 4 | 0 | 0 | 4 |
| Sweet potato, cooked | 72 | 0 | 5 | 0 | 77 |
| Sweet potato, unspecified | 0 | 11 | 0 | 10 | 21 |
| Textured vegetable protein, cooked | 1 | 0 | 0 | 0 | 1 |
| Tilapia, cooked | 4 | 0 | 1 | 0 | 5 |
| Tofu, unspecified | 1 | 0 | 0 | 0 | 1 |
| Tomato, raw | 4 | 1 | 0 | 3 | 8 |
| Tuna meal, unspecified | 1 | 0 | 0 | 0 | 1 |
| Tuna pulp, unspecified | 0 | 2 | 0 | 13 | 15 |
| Turkey meal, unspecified | 0 | 14 | 0 | 3 | 17 |
| Turkey meat, unspecified | 8 | 3 | 0 | 2 | 13 |
| Turnip, cooked | 2 | 0 | 0 | 0 | 2 |
| Wheat bran, unspecified | 7 | 90 | 0 | 49 | 146 |
| Wheat flour, unspecified | 8 | 47 | 0 | 68 | 123 |
| Wheat germ, unspecified | 7 | 0 | 0 | 0 | 7 |
| Wheat gluten, unspecified | 1 | 69 | 0 | 66 | 136 |
| Whey protein concentrate, unspecified | 0 | 1 | 0 | 0 | 1 |
| White bread, unspecified | 4 | 0 | 2 | 0 | 6 |
| White rice, cooked | 43 | 0 | 6 | 0 | 49 |
| Whole chicken, cooked | 0 | 15 | 0 | 16 | 31 |
| Whole cornmeal, unspecified | 0 | 216 | 0 | 139 | 355 |
| Whole milk, unspecified | 2 | 0 | 1 | 0 | 3 |
| Whole oats, raw | 0 | 52 | 0 | 23 | 75 |
| Whole wheat flour, unspecified | 4 | 0 | 0 | 0 | 4 |
| Whole wheat, unspecified | 0 | 18 | 0 | 7 | 25 |
| Yam, cooked | 20 | 0 | 4 | 0 | 24 |
| Yogurt, unspecified | 8 | 0 | 5 | 0 | 13 |
| Yucca flour, unspecified | 0 | 16 | 0 | 12 | 28 |
| Yucca starch, unspecified | 7 | 0 | 3 | 0 | 10 |
| Yucca, cooked | 7 | 0 | 0 | 0 | 7 |
| Zucchini, cooked | 67 | 0 | 7 | 0 | 74 |

**Table S4.** Estimated environmental impact per 1000 kcal of ingredients for greenhouse gas emission, land use, acidifying emission, eutrophying emission, freshwater withdrawal, and stress-weighted water use.

|  | Land use (m^2^/1000 kcal) | Co_2_eq emission (kg/1000 kcal) | SO_2_eq emission (g/1000 kcal) | PO_4_^3-^eq (g/1000 kcal) | Freshwater withdrawal (L/1000 kcal) | Stress-weighted water use (L/1000 kcal) |
| --- | --- | --- | --- | --- | --- | --- |
| Apple pomace, unspecified^1^ | 0.39 | 0.26 | 2.26 | 0.97 | 116.20 | 8359.05 |
| Apple, raw^2^ | 0.80 | 1.20 | 7.40 | 10.00 | 610.00 | 13450.00 |
| Arracacha, cooked^3^ | 0.03 | 0.05 | 0.33 | 0.18 | 3.15 | 104.51 |
| Artichoke, raw^2^ | 0.75 | 0.94 | 12.08 | 4.34 | 194.34 | 9266.04 |
| Bacon, cooked^2^ | 285.83 | 87.14 | 278.86 | 263.17 | 1268.80 | 30358.83 |
| Banana, raw^2^ | 2.13 | 1.01 | 7.19 | 3.71 | 129.21 | 743.82 |
| Barley, cooked^2^ | 0.37 | 0.41 | 0.44 | 0.17 | 1.01 | 47.02 |
| Barley, unspecified^4^ | 0.20 | 0.22 | 0.24 | 0.09 | 0.55 | 25.54 |
| Beef heart, cooked^2^ | 283.07 | 86.30 | 276.17 | 260.64 | 1256.57 | 30066.25 |
| Beef heart, raw^2^ | 259.47 | 79.11 | 253.14 | 238.90 | 1151.80 | 27559.35 |
| Beef kidney, cooked^2^ | 328.00 | 100.00 | 320.00 | 302.00 | 1456.00 | 34838.00 |
| Beef kidney, raw^4^ | 288.24 | 87.88 | 281.21 | 265.39 | 1279.52 | 30615.21 |
| Beef liver, cooked^2^ | 320.37 | 97.67 | 312.56 | 294.98 | 1422.14 | 34027.81 |
| Beef liver, raw^2^ | 326.99 | 99.69 | 319.01 | 301.07 | 1451.51 | 34730.53 |
| Beef lung, cooked^2^ | 371.13 | 113.15 | 362.08 | 341.71 | 1647.46 | 39419.14 |
| Beef lung, raw^2^ | 288.78 | 88.04 | 281.74 | 265.89 | 1281.91 | 30672.59 |
| Beef meal, unspecified^1^ | 325.05 | 99.10 | 317.12 | 299.28 | 1442.88 | 34524.14 |
| Beef meat, cooked^2^ | 231.67 | 70.63 | 226.02 | 213.31 | 1028.40 | 24606.77 |
| Beef meat, unspecified^4^ | 161.10 | 49.12 | 157.17 | 148.33 | 715.12 | 17110.82 |
| Beef offal, unspecified^4^ | 301.49 | 91.92 | 294.13 | 277.59 | 1338.30 | 32021.85 |
| Beef shoulder clod, cooked^2^ | 236.99 | 72.25 | 231.21 | 218.20 | 1052.00 | 25171.41 |
| Beef spleen, cooked^2^ | 19.17 | 13.25 | 153.37 | 81.91 | 1934.57 | 72027.06 |
| Beef spleen, raw^4^ | 283.89 | 86.55 | 276.97 | 261.39 | 1260.19 | 30152.89 |
| Beef tallow, unspecified^4^ | 0.00 | 0.00 | 0.00 | 0.00 | 0.00 | 0.00 |
| Beef tongue, cooked^2^ | 142.61 | 43.48 | 139.13 | 131.30 | 633.04 | 15146.96 |
| Beef tongue, raw^2^ | 109.09 | 33.26 | 106.43 | 100.44 | 484.25 | 11586.75 |
| Beet pulp, unspecified^4^ | 0.13 | 0.17 | 1.25 | 0.69 | 12.09 | 401.12 |
| Beets, cooked^2^ | 0.11 | 0.15 | 1.11 | 0.61 | 10.69 | 354.71 |
| Bell pepper, cooked^2^ | 1.43 | 1.79 | 22.86 | 8.21 | 367.86 | 17539.29 |
| Blueberry, unspecified^2^ | 4.21 | 2.63 | 21.05 | 10.70 | 736.84 | 37126.32 |
| Boar meat, unspecified^2^ | 19.39 | 13.40 | 155.15 | 82.87 | 1957.06 | 72864.24 |
| Borage seed oil, unspecified^2^ | 1.22 | 0.70 | 1.78 | 1.33 | 46.11 | 1654.22 |
| Brazilian nut, raw^2^ | 0.76 | 0.26 | 1.87 | 1.17 | 153.85 | 5129.34 |
| Brewer's rice^4^ | 0.80 | 1.20 | 7.40 | 10.00 | 610.00 | 13450.00 |
| Brewer's yeast, unspecified^4^ | 0.00 | 0.00 | 0.00 | 0.00 | 0.00 | 0.00 |
| Broccoli leaves, cooked^2^ | 3.94 | 6.39 | 8.73 | 5.32 | 126.65 | 8998.54 |
| Broccoli, cooked^2^ | 2.52 | 4.08 | 5.58 | 3.40 | 80.92 | 5749.40 |
| Broken bean, unspecified^4^ | 5.48 | 0.60 | 7.51 | 6.00 | 153.11 | 7879.85 |
| Broken rice, unspecified^4^ | 0.80 | 1.20 | 7.40 | 10.00 | 610.00 | 13450.00 |
| Brown rice, cooked^2^ | 0.80 | 1.20 | 7.40 | 10.00 | 610.00 | 13450.00 |
| Butter, unspecified^2^ | 0.21 | 0.06 | 0.40 | 0.24 | 13.40 | 432.23 |
| Cabbage, cooked^2^ | 2.04 | 3.31 | 4.53 | 2.76 | 65.71 | 4668.63 |
| Canola oil, unspecified^2^ | 1.27 | 0.73 | 1.78 | 1.33 | 46.11 | 1654.22 |
| Carrot, cooked^2^ | 0.86 | 1.14 | 8.29 | 4.57 | 80.00 | 2654.29 |
| Carrot, unspecified | 0.73 | 0.98 | 7.07 | 3.90 | 68.29 | 2265.85 |
| Cauliflower, cooked^2^ | 2.96 | 4.80 | 6.56 | 4.00 | 95.20 | 6764.00 |
| Celery, raw^2^ | 2.50 | 3.13 | 40.00 | 14.38 | 643.75 | 30693.75 |
| Cellulose, unspecified^4^ | 0.00 | 0.00 | 0.00 | 0.00 | 0.00 | 0.00 |
| Chard, cooked^2^ | 0.91 | 1.14 | 14.55 | 5.23 | 234.09 | 11161.36 |
| Chayote, cooked^2^ | 1.67 | 2.08 | 26.67 | 9.58 | 429.17 | 20462.50 |
| Cheese curds, unspecified^2^ | 45.39 | 12.48 | 85.10 | 51.06 | 2880.99 | 92939.37 |
| Chia seeds, raw^2^ | 2.48 | 0.27 | 3.40 | 2.72 | 69.43 | 3573.12 |
| Chicken breast, cooked^2^ | 13.63 | 10.94 | 113.23 | 53.74 | 731.22 | 15710.61 |
| Chicken breast, raw^2^ | 13.31 | 10.69 | 110.63 | 52.50 | 714.38 | 15348.75 |
| Chicken breast, unspecified^2^ | 13.31 | 10.69 | 110.63 | 52.50 | 714.38 | 15348.75 |
| Chicken drumsticks, cooked^2^ | 11.55 | 9.28 | 96.01 | 45.56 | 619.97 | 13320.41 |
| Chicken gizzard, cooked^2^ | 14.01 | 11.25 | 116.43 | 55.25 | 751.86 | 16154.06 |
| Chicken gizzard, raw^2^ | 13.34 | 10.71 | 110.84 | 52.60 | 715.79 | 15379.23 |
| Chicken liver hydrolysate, unspecified^4^ | 12.62 | 10.13 | 104.89 | 49.78 | 677.33 | 14552.89 |
| Chicken liver, cooked^2^ | 10.40 | 8.35 | 86.42 | 41.01 | 558.04 | 11989.79 |
| Chicken meat, cooked^2^ | 8.00 | 6.42 | 66.49 | 31.55 | 429.36 | 9225.14 |
| Chicken meat, raw^4^ | 8.00 | 6.42 | 66.49 | 31.55 | 429.36 | 9225.14 |
| Chicken neck, raw^2^ | 5.64 | 4.53 | 46.84 | 22.23 | 302.49 | 6499.09 |
| Chicken protein hydrolysate, unpecified^4^ | 17.46 | 14.02 | 145.08 | 68.85 | 936.89 | 20129.51 |
| Chickpea, cooked^2^ | 3.94 | 0.43 | 5.40 | 4.32 | 110.21 | 5672.02 |
| Chinese cabbage, cooked^2^ | 2.86 | 3.57 | 45.71 | 16.43 | 735.71 | 35078.57 |
| Coconut oil, unspecified^2^ | 1.28 | 0.73 | 1.78 | 1.33 | 46.11 | 1654.22 |
| Coconut pulp, unspecified^1^ | 0.37 | 0.45 | 2.38 | 0.98 | 63.11 | 3906.97 |
| Coconut, raw^2^ | 0.15 | 0.19 | 0.98 | 0.41 | 26.01 | 1610.30 |
| Cod live roil, unspecified^2^ | 0.99 | 1.60 | 3.22 | 11.44 | 179.89 | 2025.44 |
| Collagen, unspecified^4^ | 468.57 | 142.86 | 457.14 | 431.43 | 2080.00 | 49768.57 |
| Collards, cooked^2^ | 3.02 | 4.90 | 6.70 | 4.08 | 97.18 | 6904.92 |
| Corn germ, unspecified^4^ | 0.70 | 0.40 | 2.60 | 0.90 | 48.00 | 2402.00 |
| Corn gluten meal 21, unspecified^4^ | 0.70 | 0.40 | 2.60 | 0.90 | 48.00 | 2402.00 |
| Corn gluten meal 60, unspecified^4^ | 0.70 | 0.40 | 2.60 | 0.90 | 48.00 | 2402.00 |
| Corn oil, unspecified^2^ | 2.08 | 0.42 | 3.11 | 5.67 | 112.00 | 4041.00 |
| Cornmeal, unspecified^2^ | 0.70 | 0.40 | 2.60 | 0.90 | 48.00 | 2402.00 |
| Defatted rice bran, unspecified^4^ | 0.80 | 1.20 | 7.40 | 10.00 | 610.00 | 13450.00 |
| Dextrose, unspecified^2^ | 0.00 | 0.00 | 0.00 | 0.00 | 0.00 | 0.00 |
| Duck meat, cooked^2^ | 4.00 | 3.21 | 33.25 | 15.78 | 214.69 | 4612.82 |
| Duck meat, raw^2^ | 2.02 | 1.62 | 16.78 | 7.96 | 108.36 | 2328.15 |
| Egg powder, unspecified^4^ | 5.16 | 3.80 | 43.42 | 18.09 | 471.26 | 14658.69 |
| Egg, cooked^2^ | 4.63 | 3.41 | 38.96 | 16.23 | 422.85 | 13153.00 |
| Eggplant, cooked^2^ | 1.14 | 1.43 | 18.29 | 6.57 | 294.29 | 14031.43 |
| Escarole, cooked^2^ | 1.74 | 2.17 | 27.83 | 10.00 | 447.83 | 21352.17 |
| Farro, cooked^2^ | 3.84 | 0.42 | 5.26 | 4.21 | 107.37 | 5525.79 |
| Fish meal, unspecified^4^ | 8.56 | 13.88 | 67.09 | 238.28 | 3745.45 | 42171.57 |
| Fish oil, unspecified^4^ | 0.00 | 0.00 | 0.00 | 0.00 | 0.00 | 0.00 |
| Fish protein hydrolysate, unspecified^4^ | 9.10 | 14.75 | 71.31 | 253.28 | 3981.15 | 44825.41 |
| Flaxseed oil, unspecified^2^ | 1.27 | 0.73 | 1.78 | 1.33 | 46.11 | 1654.22 |
| Flaxseed, unspecified^4^ | 3.55 | 0.39 | 4.86 | 3.89 | 99.24 | 5107.31 |
| Garlic, raw^2^ | 0.28 | 0.35 | 2.52 | 2.24 | 9.79 | 651.75 |
| Green beans, cooked^2^ | 1.14 | 1.43 | 18.29 | 6.57 | 294.29 | 14031.43 |
| Hake, cooked^2^ | 8.07 | 13.08 | 63.23 | 224.57 | 3529.95 | 39745.20 |
| Hemoglobin powder, unspecified^4^ | 468.57 | 142.86 | 457.14 | 431.43 | 2080.00 | 49768.57 |
| Herring, raw^2^ | 4.21 | 6.82 | 32.96 | 117.08 | 1840.33 | 20721.07 |
| Lamb meal, unspecified^4^ | 345.50 | 37.35 | 128.86 | 91.51 | 1682.68 | 132461.29 |
| Lamb meat and bone meal, unspecified^4^ | 369.18 | 39.91 | 137.69 | 97.78 | 1798.00 | 141539.47 |
| Lamb meat, cooked^2^ | 154.29 | 16.68 | 57.55 | 40.87 | 751.45 | 59154.08 |
| Lamb meat, raw^2^ | 116.96 | 12.64 | 43.62 | 30.98 | 569.62 | 44840.74 |
| Lamb offal, unspecified^4^ | 247.77 | 26.79 | 92.41 | 65.63 | 1206.70 | 94991.52 |
| Leek, cooked^2^ | 1.29 | 1.61 | 11.61 | 10.32 | 45.16 | 3006.45 |
| Lentil, cooked^2^ | 5.68 | 0.62 | 7.78 | 6.22 | 158.63 | 8163.88 |
| Lignocellulose, unspecified^4^ | 0.00 | 0.00 | 0.00 | 0.00 | 0.00 | 0.00 |
| Meat and bone meal, unspecified^4^ | 327.27 | 99.78 | 319.29 | 301.33 | 1452.77 | 34760.75 |
| Mechanically separated beef meat, unspecified^4^ | 68.99 | 21.03 | 67.30 | 63.52 | 306.24 | 7327.37 |
| Mechanically separated chicken meat, unspecified^4^ | 3.49 | 2.80 | 29.02 | 13.77 | 187.41 | 4026.56 |
| Milk cream, unspecified^2^ | 0.75 | 0.27 | 1.67 | 0.92 | 52.46 | 1652.71 |
| Millet, cooked^2^ | 2.15 | 0.24 | 2.95 | 2.36 | 60.17 | 3096.76 |
| Mozzarella, unspecified^2^ | 29.56 | 8.13 | 55.43 | 33.26 | 1876.32 | 60529.27 |
| Oat bran, raw^2^ | 7.60 | 2.50 | 4.10 | 4.30 | 184.00 | 7162.00 |
| Oat flour, unspecified^2^ | 7.61 | 12.35 | 59.67 | 211.93 | 3331.28 | 37508.23 |
| Okra, cooked^2^ | 1.82 | 2.27 | 29.09 | 10.45 | 468.18 | 22322.73 |
| Olive oil, unspecified^2^ | 3.00 | 0.62 | 4.22 | 4.11 | 238.00 | 19720.00 |
| Onion, raw^2^ | 1.00 | 1.25 | 9.00 | 8.00 | 35.00 | 2330.00 |
| Ostrich meat, raw^2^ | 8.70 | 6.99 | 72.30 | 34.31 | 466.90 | 10031.57 |
| Papaya, raw^2^ | 2.09 | 2.56 | 13.49 | 5.58 | 358.14 | 22169.77 |
| Parboiled rice, cooked^2^ | 0.80 | 1.20 | 7.40 | 10.00 | 610.00 | 13450.00 |
| Pea flour, unspecified^4^ | 1.53 | 13.79 | 2.91 | 2.60 | 136.36 | 9635.68 |
| Pea hull, unspecified^4^ | 4.46 | 40.14 | 8.47 | 7.58 | 396.95 | 28049.53 |
| Pea, cooked^2^ | 1.41 | 12.72 | 2.69 | 2.40 | 125.81 | 8889.87 |
| Pea, unspecified^4^ | 1.48 | 13.31 | 2.81 | 2.51 | 131.58 | 9297.91 |
| Peanut butter, unspecified^2^ | 1.30 | 0.45 | 3.19 | 2.01 | 262.95 | 8767.01 |
| Plasma powder, unspecified^4^ | 438.17 | 133.59 | 427.48 | 403.44 | 1945.04 | 46539.31 |
| Pork ham, cooked^2^ | 12.31 | 8.50 | 98.47 | 52.59 | 1242.05 | 46243.49 |
| Pork ham, raw^2^ | 7.83 | 5.41 | 62.61 | 33.44 | 789.69 | 29401.21 |
| Pork heart, cooked^2^ | 17.54 | 12.12 | 140.32 | 74.95 | 1770.00 | 65899.81 |
| Pork kidney, cooked^2^ | 31.43 | 21.71 | 251.43 | 134.29 | 3171.43 | 118077.14 |
| Pork liver, unspecified^4^ | 23.04 | 15.92 | 184.29 | 98.43 | 2324.61 | 86548.69 |
| Pork loin, cooked^2^ | 15.08 | 10.42 | 120.65 | 64.44 | 1521.83 | 56659.92 |
| Pork lung, cooked^2^ | 26.48 | 18.30 | 211.85 | 113.15 | 2672.22 | 99490.93 |
| Pork meat and bone meal, unspecified^4^ | 21.95 | 15.17 | 175.61 | 93.79 | 2215.08 | 82470.73 |
| Pork offal, unspecified^4^ | 21.29 | 14.71 | 170.31 | 90.96 | 2148.24 | 79982.18 |
| Pork protein isolate, unspecified^4^ | 21.15 | 14.62 | 169.23 | 90.38 | 2134.62 | 79475.00 |
| Pork tallow, unspecified^4^ | 0.00 | 0.00 | 0.00 | 0.00 | 0.00 | 0.00 |
| Pork tenderloin, cooked^2^ | 20.13 | 13.91 | 161.05 | 86.01 | 2031.38 | 75631.30 |
| Pork tenderloin, raw^2^ | 18.93 | 13.08 | 151.43 | 80.88 | 1910.13 | 71116.88 |
| Potato starch, unspecified^4^ | 1.20 | 0.60 | 5.30 | 4.80 | 81.00 | 3763.00 |
| Potato, cooked^2^ | 1.20 | 0.60 | 5.30 | 4.80 | 81.00 | 3763.00 |
| Poultry by-product meal, unspecified^4^ | 14.04 | 11.27 | 116.64 | 55.35 | 753.21 | 16183.20 |
| Poultry fat, unspecified^4^ | 0.00 | 0.00 | 0.00 | 0.00 | 0.00 | 0.00 |
| Poultry liver, unspecified^4^ | 13.25 | 10.64 | 110.09 | 52.24 | 710.90 | 15274.17 |
| Poultry offal, unspecified^4^ | 5.53 | 4.44 | 45.91 | 21.79 | 296.50 | 6370.43 |
| Powdered milk, unspecified^2^ | 4.60 | 1.64 | 10.22 | 5.62 | 320.95 | 10111.96 |
| Pumpkin seed, toasted^2^ | 0.07 | 0.09 | 1.11 | 0.40 | 17.94 | 855.57 |
| Quick oats, raw^2^ | 7.60 | 2.50 | 4.10 | 4.30 | 184.00 | 7162.00 |
| Quinoa, cooked^2^ | 3.42 | 0.38 | 4.69 | 3.75 | 95.68 | 4924.46 |
| Quinoa, unspecified^4^ | 3.42 | 0.38 | 4.69 | 3.75 | 95.68 | 4924.46 |
| Rabbit meat, raw^2^ | 10.47 | 8.40 | 86.98 | 41.28 | 561.69 | 12068.33 |
| Rabbit meat, unspecified^2^ | 10.47 | 8.40 | 86.98 | 41.28 | 561.69 | 12068.33 |
| Rice flour, unspecified^2^ | 0.80 | 1.20 | 7.40 | 10.00 | 610.00 | 13450.00 |
| Rice noodle, cooked^2^ | 1.15 | 0.77 | 6.73 | 2.88 | 346.15 | 24901.92 |
| Ricotta, unspecified^2^ | 36.00 | 9.90 | 67.50 | 40.50 | 2285.10 | 73716.30 |
| Salmon meal, unspecified^4^ | 6.62 | 10.73 | 51.86 | 184.18 | 2895.05 | 32596.56 |
| Salmon pulp, unspecified^4^ | 4.53 | 7.34 | 35.47 | 125.99 | 1980.43 | 22298.47 |
| Salmon, cooked^2^ | 3.97 | 6.44 | 31.11 | 110.50 | 1736.89 | 19556.35 |
| Sardine pulp, unspecified^4^ | 3.08 | 5.00 | 24.17 | 85.83 | 1349.17 | 15190.83 |
| Sardine, cooked^2^ | 7.85 | 12.73 | 61.52 | 218.48 | 3434.24 | 38667.58 |
| Sardine, unspecified^4^ | 7.85 | 12.73 | 61.52 | 218.48 | 3434.24 | 38667.58 |
| Sesame seed oil, unspecified^2^ | 1.27 | 0.73 | 1.78 | 1.33 | 46.11 | 1654.22 |
| Shrimp meal, unspecified^4^ | 2.90 | 26.09 | 130.43 | 223.19 | 3449.28 | 124869.57 |
| Shrimp, cooked^2^ | 4.84 | 43.60 | 218.00 | 373.02 | 5764.89 | 208698.67 |
| Sorghum, unspecified^4^ | 2.25 | 0.25 | 3.08 | 2.47 | 62.90 | 3237.01 |
| Soy bran, unspecified^4^ | 12.58 | 1.38 | 17.23 | 13.79 | 351.59 | 18094.87 |
| Soy fiber, unspecified^4^ | 4.59 | 0.50 | 6.28 | 5.02 | 128.14 | 6594.68 |
| Soy flour, unspecified^2^ | 10.66 | 1.17 | 14.60 | 11.68 | 297.83 | 15327.92 |
| Soy hull, unspecified^4^ | 4.59 | 0.50 | 6.28 | 5.02 | 128.14 | 6594.68 |
| Soy protein concentrate, unspecified^4^ | 15.81 | 1.73 | 21.65 | 17.32 | 441.72 | 22733.35 |
| Soy protein hydrolysate, unspecified^4^ | 17.78 | 1.95 | 24.35 | 19.48 | 496.80 | 25568.34 |
| Soy protein isolate, unspecified^4^ | 17.40 | 1.91 | 23.84 | 19.07 | 486.29 | 25027.41 |
| Soy, cooked^2^ | 7.73 | 0.85 | 10.59 | 8.47 | 215.98 | 11115.51 |
| Soybean oil, unspecified^4^ | 1.22 | 0.70 | 1.78 | 1.33 | 46.11 | 1654.22 |
| Spaghetti, cooked^2^ | 0.60 | 1.40 | 5.00 | 2.70 | 242.00 | 12479.00 |
| Spinach, cooked^2^ | 1.74 | 2.17 | 27.83 | 10.00 | 447.83 | 21352.17 |
| Squash, cooked^2^ | 1.08 | 1.35 | 17.30 | 6.22 | 278.38 | 13272.97 |
| Squash, unspecified^2^ | 1.18 | 1.47 | 18.82 | 6.76 | 302.94 | 14444.12 |
| Squid meal, unspecified^4^ | 5.07 | 45.59 | 227.93 | 390.02 | 6027.54 | 218207.03 |
| Sugarcane fiber, unspecified^2^ | 0.00 | 0.00 | 0.00 | 0.00 | 0.00 | 0.00 |
| Sunflower oil, unspecified^2^ | 2.08 | 0.42 | 3.11 | 5.67 | 112.00 | 4041.00 |
| Sunflower seed, toasted^2^ | 0.07 | 0.09 | 1.10 | 0.40 | 17.70 | 843.81 |
| Sweet potato flour, unspecified^2^ | 1.20 | 0.60 | 5.30 | 4.80 | 81.00 | 3763.00 |
| Sweet potato, cooked^2^ | 1.20 | 0.60 | 5.30 | 4.80 | 81.00 | 3763.00 |
| Sweet potato, unspecified^2^ | 1.20 | 0.60 | 5.30 | 4.80 | 81.00 | 3763.00 |
| Textured vegetable protein, cooked^2^ | 11.49 | 1.26 | 15.74 | 12.59 | 321.03 | 16522.28 |
| Tilapia, cooked^2^ | 7.56 | 12.26 | 59.25 | 210.43 | 3307.57 | 37241.28 |
| Tofu, unspecified^2^ | 7.76 | 0.85 | 10.63 | 8.51 | 216.88 | 11162.09 |
| Tomato, raw^2^ | 14.76 | 5.62 | 119.47 | 52.71 | 2600.14 | 37498.24 |
| Tuna meal, unspecified^4^ | 7.60 | 2.50 | 4.10 | 4.30 | 184.00 | 7162.00 |
| Tuna pulp, unspecified^4^ | 5.47 | 8.86 | 42.84 | 152.16 | 2391.70 | 26929.20 |
| Turkey meal, unspecified^4^ | 12.42 | 9.97 | 103.19 | 48.97 | 666.39 | 14317.72 |
| Turkey meat, cooked^2^ | 9.57 | 7.69 | 79.55 | 37.75 | 513.69 | 11036.99 |
| Turkey meat, unspecified^2^ | 9.43 | 7.57 | 78.37 | 37.19 | 506.11 | 10874.11 |
| Turnip, cooked^2^ | 0.10 | 0.13 | 0.94 | 0.52 | 9.04 | 299.81 |
| Wheat bran, unspecified^4^ | 0.60 | 1.40 | 5.00 | 2.70 | 242.00 | 12479.00 |
| Wheat flour, unspecified^4^ | 0.60 | 1.40 | 5.00 | 2.70 | 242.00 | 12479.00 |
| Wheat germ, unspecified^4^ | 0.60 | 1.40 | 5.00 | 2.70 | 242.00 | 12479.00 |
| Wheat gluten, unspecified^4^ | 0.60 | 1.40 | 5.00 | 2.70 | 242.00 | 12479.00 |
| Whey protein concentrate, unspecified^1^ | 79.20 | 28.16 | 176.00 | 96.80 | 5526.40 | 174116.80 |
| White bread, unspecified^2^ | 0.60 | 1.40 | 5.00 | 2.70 | 242.00 | 12479.00 |
| White rice, cooked^2^ | 0.80 | 1.20 | 7.40 | 10.00 | 610.00 | 13450.00 |
| Whole chicken, cooked^2^ | 8.00 | 6.42 | 66.49 | 31.55 | 429.36 | 9225.14 |
| Whole cornmeal, unspecified^4^ | 0.70 | 0.40 | 2.60 | 0.90 | 48.00 | 2402.00 |
| Whole milk, unspecified^2^ | 4.65 | 1.65 | 10.33 | 5.68 | 324.30 | 10217.36 |
| Whole oats, raw^4^ | 7.60 | 2.50 | 4.10 | 4.30 | 184.00 | 7162.00 |
| Whole wheat flour, unspecified^2^ | 0.60 | 1.40 | 5.00 | 2.70 | 242.00 | 12479.00 |
| Whole wheat, unspecified^4^ | 0.60 | 1.40 | 5.00 | 2.70 | 242.00 | 12479.00 |
| Yam, cooked^2^ | 0.04 | 0.05 | 0.37 | 0.21 | 3.60 | 119.33 |
| Yogurt, unspecified^2^ | 5.12 | 1.82 | 11.38 | 6.26 | 357.24 | 11255.31 |
| Yucca flour, unspecified^4^ | 1.90 | 1.40 | 3.50 | 0.70 | 0.00 | 0.00 |
| Yucca starch, unspecified^4^ | 1.90 | 1.40 | 3.50 | 0.70 | 0.00 | 0.00 |
| Yucca, cooked^2^ | 0.30 | 0.40 | 2.90 | 1.60 | 28.00 | 929.00 |
| Zucchini, cooked^2^ | 2.67 | 3.33 | 42.67 | 15.33 | 686.67 | 32740.00 |

Legend: ^1^Composition from Butolo [1]; ^2^composition from the Brazilian Table of Food Composition [2]; ^3^composition from the Brazilian Association of the Pet Food Industry (ABINPET) [3]; ^4^composition from USDA’s FoodData Central [4].

**References:**

1. Butolo, J. E. *Qualidade de ingredientes na alimentação animal*. (Mundo Agro Editora, 2010).

2. TACO. *Tabela Brasileira de Composição de Alimentos*. (NEPA-UNICAMP, 2011).

3. ABINPET. *Manual Pet Food Brasil*. (Centrografica, 2017).

4. USDA. Food Data Central. *Food Data Central* https://fdc.nal.usda.gov/ (2020).
